# Cage Confinement Strategy in Perovskite-QDs@MOF for Boosting the Third-Order Nonlinear Optical Performance

**DOI:** 10.1021/acscentsci.5c00863

**Published:** 2025-07-23

**Authors:** Yupei Sun, Kangshuai Geng, Jing Huang, Yi Wei, Hongwei Hou

**Affiliations:** College of Chemistry, 12636 Zhengzhou University, Zhengzhou, Henan 450001, China

## Abstract

Perovskite quantum dots (PeQDs) exhibit great potential in third-order nonlinear optics due to their unique optical properties and nanoscale dimensions. However, technical challenges still exist in controlling the size, morphology, and distribution. To address this issue, we utilize the confinement effect of the Cu-metal–organic framework (MOF) ({[Cu_1.75_L_0.75_(Pz-NH_2_)_0.125_(μ_3_-O)_0.125_(μ_2_-OH)_0.25_(H_2_O)_0.375_]·3CH_3_CN}_*n*_, where L = 5,5′-(1*H*-2,3,5-triazole-1,4-diyl)­diisophthalic acid) with cage-like pores to encapsulate ABBr_3_-QDs (A = MA (methylammonium), FA (formamidine); B = Pb, Sn), resulting in uniformly dispersed ABBr_3_-QDs within the Cu-MOF. The results of the third-order nonlinear optical (NLO) response show that, compared to PeQDs, the third-order NLO absorption of ABBr_3_-QDs@Cu-MOF is enhanced by a factor of 6.36. Theoretical calculations and femtosecond transient absorption spectroscopy (fs-TAS) analysis reveal that the observed changes in NLO properties are primarily attributed to the redistribution of the electron clouds in PeQDs and Cu-MOF. This reconfiguration alters the band structure, facilitates the separation of free electron–hole pairs, and precisely controls the direction and relaxation time of free carrier transport, leading to a significant improvement in third-order NLO performance. Furthermore, by adjusting the composition of the cations, the third-order NLO signal of PeQDs@Cu-MOF can be effectively tuned. This study provides new insights into the development of high-performance third-order NLO materials.

## Graphical abstract

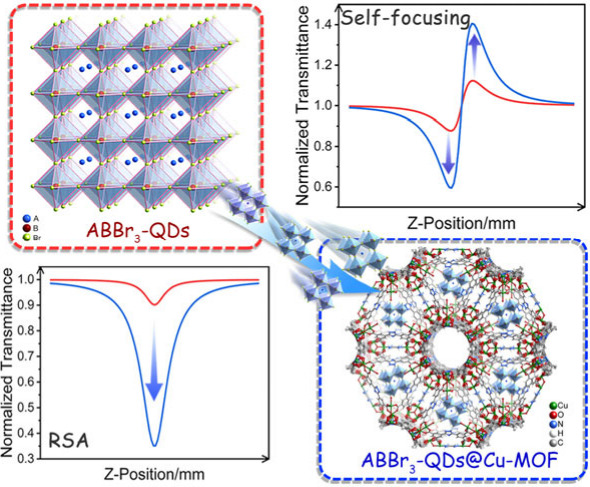

## Introduction

Organic–inorganic halide perovskite quantum dots (PeQDs), as a new class of nanomaterials, have garnered extensive attention in recent years for their potential in third-order nonlinear optics.^1–3^ Thanks to their outstanding optical properties, PeQDs exhibit excellent light absorption, carrier mobility, and quantum efficiency.^4,5^ These advantages enhance their electronic properties relative to bulk materials, such as the increased formation of charge pairs, energy level quantization, and a reduction in electron–phonon relaxation rates.^6–8^ Moreover, tuning the composition of PeQDs is a key strategy to further improve their optical performance. By modifying the cations in the organic and inorganic components of the perovskite, the third-order nonlinear optical (NLO) properties of the QDs can be tuned.^9–11^ Specifically, organic cations can effectively passivate the surface states of PeQDs, reducing carrier recombination and thus optimizing the material’s NLO properties.^12,13^ However, precise control over the synthesis of PeQDs remains a significant challenge, and thus, there is an urgent need to explore viable solutions.^14,15^

Encapsulating PeQDs within the pores of metal–organic frameworks (MOFs) is an effective strategy.^16^ The pore size and shape of MOFs can be rationally designed and optimized to match the size of the PeQDs, thereby enhancing the transport efficiency of electrons and holes.^17–19^ This approach not only addresses the issue of size distribution irregularities but also improves the carrier dynamics.^20–22^ The surface of PeQDs typically contains defects, which serve as recombination centers for charge carriers. By encapsulating the PeQDs within MOFs, the interaction between the internal surface of the MOF and the surface of the PeQDs helps to reduce surface defects, thereby lowering the probability of carrier recombination.^23,24^ Additionally, the electron cloud of the MOFs can interact with the electrons or holes in the PeQDs, generating a local electric field that facilitates the separation of excitons, thereby enhancing the third-order NLO response of the PeQDs.^25–27^

In this study, we use Cu-MOF with a cage-like pore structure as a template to facilitate the growth of PeQDs and confine ABBr_3_-QDs (A = MA (methylammonium), FA (formamidine); B = Pb, Sn) within the Cu-MOF to form the ABBr_3_-QDs@Cu-MOF. The third-order NLO measurements indicate that the nonlinear absorption (NLA) response of ABBr_3_-QDs@Cu-MOF is stronger than that of ABBr_3_-QDs. Specifically, under the influence of a 900 nm pulsed laser, the NLA coefficient of FAPbBr_3_-QDs@Cu-MOF is 6.36 times higher than that of FAPbBr_3_-QDs. Theoretical calculations and femtosecond transient absorption spectroscopy (fs-TAS) analyses show that the interaction between ABBr_3_-QDs and Cu-MOF alters the spatial electron cloud density and adjusts the band structure, which affects the carrier transport path and relaxation lifetime. The modulation of the electron cloud density can effectively control the nonlinear polarization of ABBr_3_-QDs@Cu-MOF, enabling the regulation of the laser phase and enhancing or altering the nonlinear refractive (NLR) signal. This finding suggests that encapsulating PeQDs within MOFs and adjusting the composition of A/B-site cations in PeQDs can effectively tune the third-order NLO properties of the material, providing new insights for the design of high-performance third-order NLO materials.

## Results and Discussion

### Synthesis and Characterization of ABBr_3_-QDs@Cu-MOF

Cu-MOF ({[Cu_1.75_L_0.75_(Pz-NH_2_)_0.125_(μ_3_-O)_0.125_(μ_2_-OH)_0.25_(H_2_O)_0.375_]·3CH_3_CN}_*n*_, where L = 5,5′-(1H-2,3,5-triazole-1,4-diyl)­diisophthalic acid) was obtained through a single-crystal-to-single-crystal transformation strategy from Co-MOF ({[Co_1.25_(HL)_0.5_(Pz-NH_2_)_0.25_(μ_3_-O)_0.25_(μ_2_-OH)_0.25_(H_2_O)]·0.125Co·0.125L·10.25H_2_O}_*n*_) (Figure S1).^28^ Single crystal X-ray diffraction reveals that the Cu-MOF crystal belongs to the hexagonal crystal system and the *P*6/*mmm* space group, and the carboxyl groups on the ligands exhibit a μ^2^-η^1^:η^1^ coordination mode. As shown in Figure S2, the asymmetric unit of the structure contains 1.75 Cu^2+^, corresponding to +3.5 charges, 0.75 L^4–^ (−3 charges), 0.125 μ_3_-O^2–^ (−0.25 charges) and 0.25 μ_2_-OH^–^ (−0.25 charges), forming an electrically neutral framework. Cu1 is coordinated with four O atoms from L^4–^, one N atom from Pz-NH_2_ and one μ_3_-O atom, while Cu2 and Cu2A are each coordinated with four O atoms from L^4–^ and one μ_3_-O, forming a trinuclear Cu cluster secondary building unit (SBU1, Cu_3_(μ_3_-O)­(O_2_C−)_6_). Cu3 is coordinated with four O atoms from L^4–^ and one terminal O atom from a H_2_O molecule, Cu4 and Cu4A are each coordinated with four O atoms from L^4–^ and one μ_2_-O, and Cu5 is coordinated with four O atoms from L^4–^ ligands, forming a tetranuclear Cu cluster secondary building unit (SBU2, Cu_4_(μ_2_-OH_2_)­(O_2_C-)_8_). The above SBUs are interconnected through L^4–^ and Pz-NH_2_, forming a three-dimensional framework with two different pore sizes. One type of pore has a size of 13.24 Å, while the other has a semiopen cage structure with a diameter of 18.00 Å. The cavity size of the Cu-MOF is well-suited to restrict the growth of PeQDs. The smaller-sized ABBr_3_-QDs can be effectively confined within the Cu-MOF cavities, with the PeQDs’ size limited to around 1.8 nm (Figure S3). PbBr_2_ was introduced into Cu-MOF through electrostatic interaction and hydrogen bonding (Br···H–N) by dispersing Cu-MOF in anhydrous DMF containing PbBr_2_, and PbBr_2_ was uniformly dispersed in the Cu-MOF cages to obtain PbBr_2_@Cu-MOF. Subsequently, PbBr_2_@Cu-MOF was added to anhydrous DMF solution of MABr, and the resulting solid was treated with toluene to finally obtain MAPbBr_3_-QDs@Cu-MOF. The Br in MAPbBr_3_-QDs readily forms hydrogen bonds with the N–H on the L^4–^. Additionally, the L^4–^ and metal cluster nodes (SBU1 and SBU2) of Cu-MOF can interact synergistically with the inorganic ions on the surface of PeQDs, effectively stabilizing the PeQDs and preventing the aggregation (Figure 1). The color change of the Cu-MOF as a whole and on the cut surface before and after loading MAPbBr_3_-QDs was recorded using a microscope. The results showed that the original Cu-MOF crystals presents a uniform green color. However, after loading MAPbBr_3_-QDs, the entire crystals and the interior turned yellowish green. This indicates that MAPbBr_3_-QDs can be uniformly dispersed in the cages of Cu-MOF (Figure S4).

**Figure 1:**
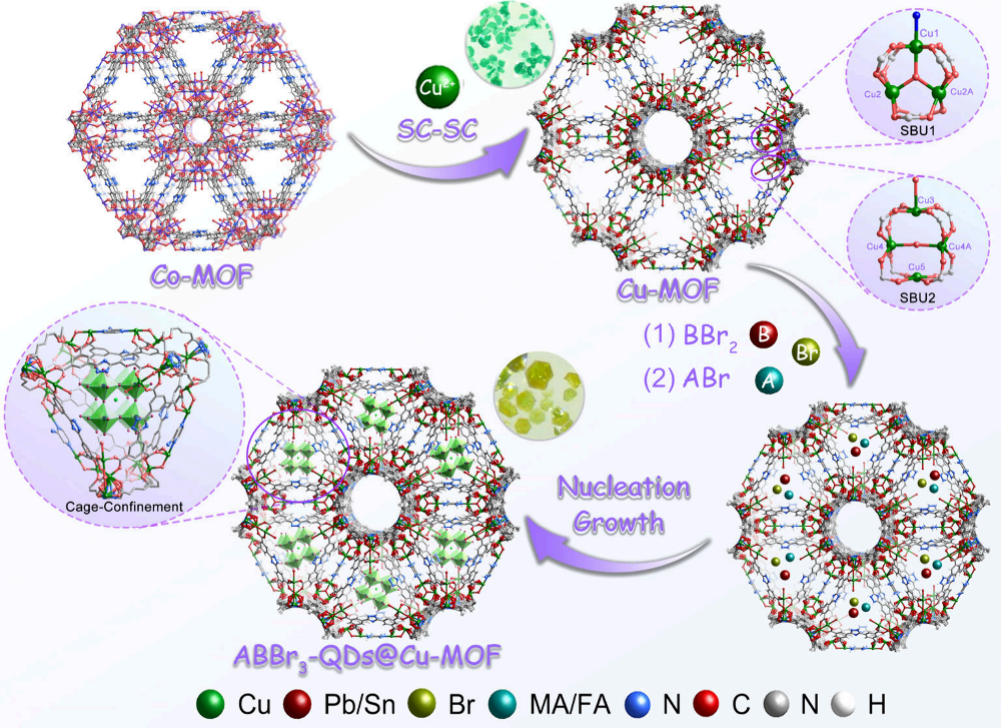
Synthesis diagram of ABBr_3_-QDs@Cu-MOF.

Using MAPbBr_3_-QDs@Cu-MOF as a representative example, various techniques were employed to investigate the internal active sites and interactions of MAPbBr_3_-QDs@Cu-MOF. The PXRD patterns reveal that the synthesized Cu-MOF exhibits peak positions consistent with those predicted by SC-XRD simulations, suggesting a high degree of crystallinity in the bulk-synthesized Cu-MOF. In MAPbBr_3_-QDs@Cu-MOF, the characteristic diffraction peaks of Cu-MOF are still observable, while distinct peaks corresponding to the MAPbBr_3_ phases are observed at 2θ = 14.7°, 20.9°, 30.3°, 33.2°, 36.8°, 42.9°, 45.8°, and 48.5° correspond to the (100), (110), (200), (210), (211), (220), (300), and (310) crystal planes, respectively (Figure 2a).^29^ These results demonstrate that the complete Cu-MOF framework facilitates the dispersion and stability of MAPbBr_3_-QDs (the PXRD patterns of other materials are shown in Figure S5).

**Figure 2:**
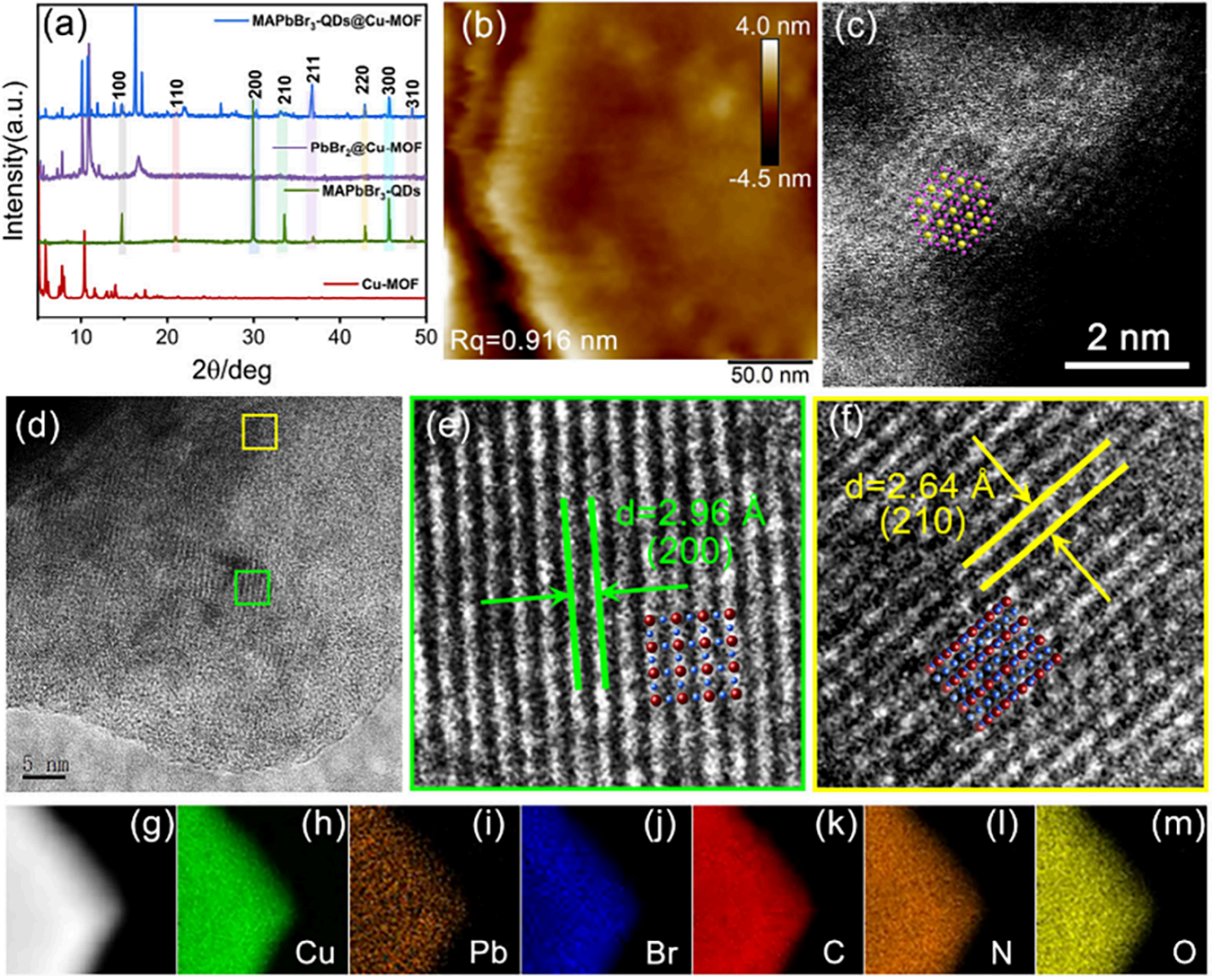
(a) PXRD patterns of Cu-MOF, MAPbBr_3_-QDs, PbBr_2_@Cu-MOF, and MAPbBr_3_-QDs@Cu-MOF; (b) AFM image of MAPbBr_3_-QDs@Cu-MOF; (c) STEM-HAADF image of MAPbBr_3_-QDs@Cu-MOF (The yellow spheres represent Pb, and the pink spheres represent Br.); (d–f) cryo-TEM images of MAPbBr_3_-QDs@Cu-MOF at different scales; (g–m) Elemental distribution images of MAPbBr_3_-QDs@Cu-MOF.

The morphology and surface information on a single MAPbBr_3_-QDs@Cu-MOF crystal were recorded using a scanning electron microscope (SEM). The results showed that MAPbBr_3_-QDs@Cu-MOF is a hexagonal prism crystal, and no deposition of MAPbBr_3_-QDs on the surface of Cu-MOF was observed. The energy dispersive spectroscopy (EDS) mapping results revealed that the elements are uniformly distributed within the crystal. The EDS line scan results showed that in the 2–10 μm region, the signals of Cu, Pb, Br, O, and N elements significantly increased and remained stable, demonstrating that these elements are mainly concentrated and uniformly distributed within the hexagonal prism crystal. The signal of the C element sharply decreased in this range, which might be related to the substrate (Figure S6). The EDS mapping images of the cross-section of MAPbBr_3_-QDs@Cu-MOF showed that the elements were uniformly dispersed throughout the crystal, ruling out the possibility of MAPbBr_3_-QDs deposition on the surface of Cu-MOF (Figure S7). Atomic force microscopy (AFM) imaging was employed to observe the surface morphology of MAPbBr_3_-QD@Cu-MOF in greater detail (Figure 2b). The surface roughness (Rq) and average surface roughness (Ra) were measured as 0.916 and 0.627 nm, respectively. The surface fluctuation range of MAPbBr_3_-QDs@Cu-MOF was observed to be between −4.5 and 4.0 nm.^30^ These results confirm that the surface of MAPbBr_3_-QDs@Cu-MOF is smooth, and there is no significant aggregation or deposition of MAPbBr_3_-QDs on the surface of Cu-MOF. We employed high-angle annular dark-field (HAADF) mode for STEM imaging (Figure 2c), which allowed us to collect scattering electrons at higher spatial angles.^31^ The STEM-HAADF images reveal the atomic arrangement of MAPbBr_3_-QDs, with yellow representing Pb atoms and pink representing Br atoms. The diameter of the formed MAPbBr_3_-QDs is approximately 1.8 nm, with their size matching the cavity height of Cu-MOF. Compared to the MAPbBr_3_-QDs (40–60 nm), the size of the QDs in MAPbBr_3_-QDs@Cu-MOF is significantly reduced (Figure S8). Furthermore, cryo-transmission electron microscopy (cryo-TEM) was used to reveal the composition and morphology of MAPbBr_3_-QDs@Cu-MOF. When the scale is 200 nm, no MAPbBr_3_-QDs were observed within the Cu-MOF (Figure S9). At a scale of 5 nm, two distinct lattice fringes of the MAPbBr_3_-QDs were observed, with interplanar spacings of 2.96 Å and 2.46 Å corresponding to the (200) and (210) crystal planes, respectively (Figure 2d–f).^32^ Elemental mapping images demonstrate a uniform distribution of all elements in both Cu-MOF and MAPbBr_3_-QDs@Cu-MOF, suggesting that MAPbBr_3_-QDs are evenly dispersed within the pores of Cu-MOF. According to the results of N_2_ adsorption–desorption isotherms, MAPbBr_3_-QDs do not exhibit physical adsorption of N_2_. Cu-MOF shows a type I isotherm, with a maximum N_2_ adsorption–desorption capacity of 263.76 cm^3^(STP) g^–1^. After introducing MAPbBr_3_-QDs into the cages of Cu-MOF, the N_2_ adsorption–desorption capacity decreases compared to Cu-MOF, with a maximum value of 127.49 cm^3^(STP) g^–1^. The significant decrease in N_2_ adsorption capacity after the introduction of MAPbBr_3_-QDs indicates that MAPbBr_3_-QDs have filled the pores of Cu-MOF. The specific surface area of Cu-MOF is 948.39 m^2^/g, while that of MAPbBr_3_-QDs@Cu-MOF is 453.67 m^2^/g, which reflects a significant impact of MAPbBr_3_-QDs on the surface area. Additionally, the pore size results confirm the loading of MAPbBr_3_-QDs in the Cu-MOF cages (Figure S10), and the activated MAPbBr_3_-QDs@Cu-MOF remains stable.

Fourier transform infrared spectroscopy was performed on the MAPbBr_3_-QDs@Cu-MOF, Cu-MOF, and MAPbBr_3_-QDs (Figure S11). Compared to MAPbBr_3_-QDs 1104.87 cm^–1^, the C–N stretching vibration of MAPbBr_3_-QDs@Cu-MOF shifts to a higher wavenumber at 1109.50 cm^–1^. Furthermore, the C=O and C–O vibration peaks in Cu-MOF exhibit significant overlap with the N–H and C–H bending vibration peaks in MAPbBr_3_-QDs. Due to the alteration in the chemical environment of the composite material, the absorption peaks of MAPbBr_3_-QDs@Cu-MOF at 1628.63 cm^–1^ and 1369.05 cm^–1^ shift relative to those in Cu-MOF (1654.22 cm^–1^ and 1365.06 cm^–1^) and MAPbBr_3_-QDs (1632.13 cm^–1^ and 1384.35 cm^–1^). This indicates that charge transfer occurs when MAPbBr_3_-QDs are encapsulated within the pores of Cu-MOF, thereby affecting the vibration frequencies of the chemical bonds. Figure 3a presents the Raman spectra of the Cu-MOF, MAPbBr_3_-QDs, and MAPbBr_3_-QDs@Cu-MOF obtained at a laser wavelength of 633 nm to minimize interference from the fluorescence background. In the Raman spectrum of MAPbBr_3_-QDs, sharp and intense bands are observed at 917.14 cm^–1^ and 970.00 cm^–1^, which corresponded to the rocking vibrations of CH_3_NH_3_^+^ and the C–N stretching vibrations, respectively. The peak at 1244.71 cm^–1^ corresponds to the rocking vibrations of CH_3_–NH_3_^+^, while the peaks at 1476.45 cm^–1^ and 1597.38 cm^–1^ correspond to the asymmetric bending and twisting vibrations of NH_3_^+^.^29,33^ In MAPbBr_3_-QDs@Cu-MOF, distinct characteristic peaks of the Cu-MOF can be observed. The peaks at 741.38 cm^–1^ and 852.23 cm^–1^ correspond to C–H vibrations, while sharp peaks at 1000.73 cm^–1^ and 1614.57 cm^–1^ are associated with C=C absorption. The double peaks at 1447.40 cm^–1^ and 1505.50 cm^–1^ correspond to the symmetric and asymmetric vibrations of O–C=O, which shift to different extents compared to the absorption peaks of the Cu-MOF (1450.48 cm^–1^ and 1503.14 cm^–1^), indicating a certain interaction between the MAPbBr_3_-QDs and the Cu-MOF.^34^ Additionally, a characteristic peak for the C–N stretching vibration of MAPbBr_3_ at 968.16 cm^–1^ can be observed in the MAPbBr_3_-QDs@Cu-MOF spectrum.

**Figure 3:**
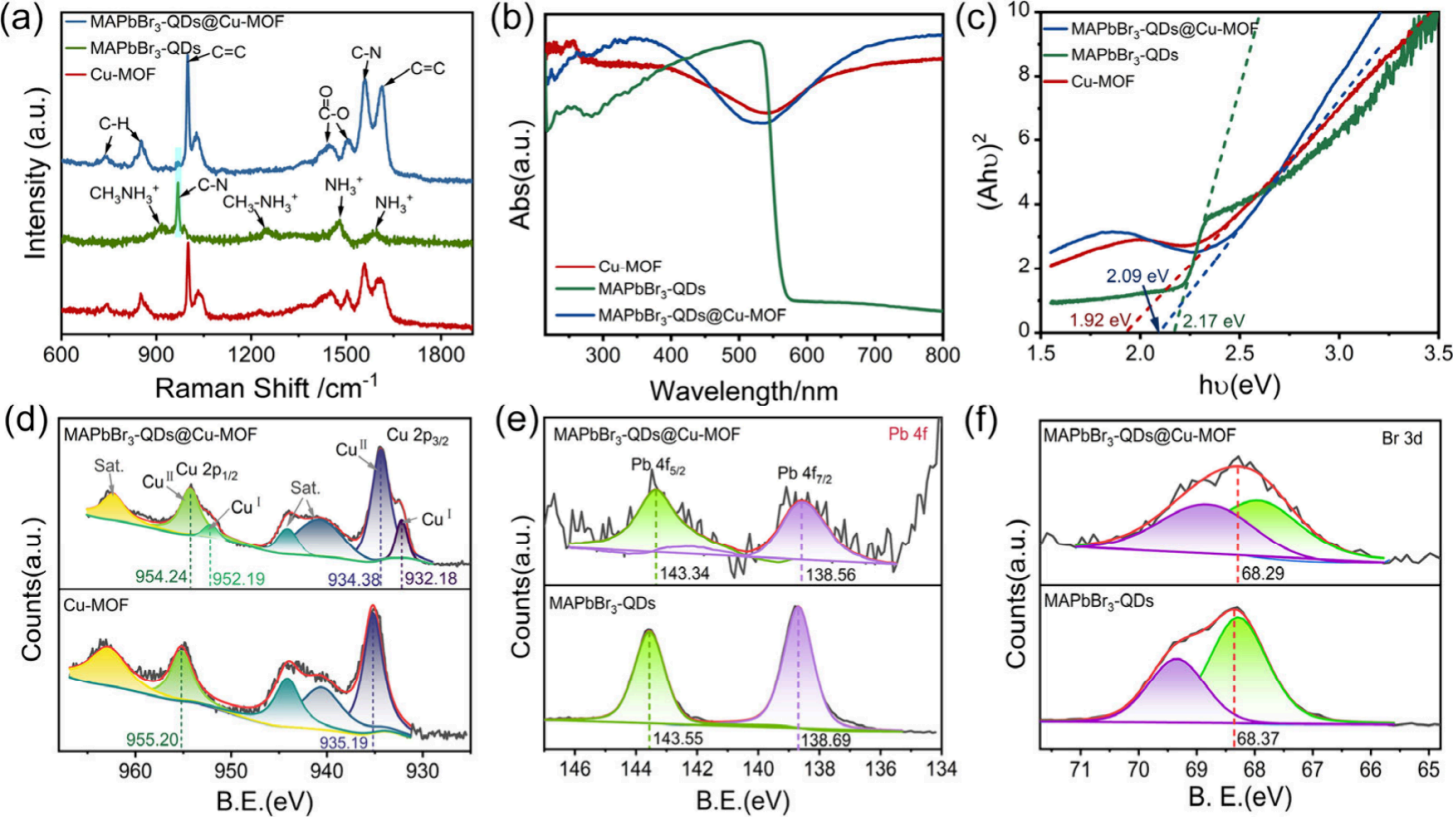
(a) Raman spectra of Cu-MOF, MAPbBr_3_-QDs, and MAPbBr_3_-QDs@Cu-MOF; (b) solid-state UV–vis diffuse reflectance spectra of Cu-MOF, MAPbBr_3_-QDs, and MAPbBr_3_-QDs@Cu-MOF; (c) the optical band gap of Cu-MOF, MAPbBr_3_-QDs, and MAPbBr_3_-QDs@Cu-MOF; (d) high-resolution Cu 2p3/2 XPS spectra of Cu-MOF and MAPbBr_3_-QDs@Cu-MOF; (e) high-resolution Pb 4f XPS spectra of MAPbBr_3_-QDs and MAPbBr_3_-QDs@Cu-MOF; (f) High-resolution Br 3d XPS spectra of MAPbBr_3_-QDs and MAPbBr_3_-QDs@Cu-MOF.

The optical absorption of MAPbBr_3_-QDs@Cu-MOF, MAPbBr_3_-QDs, and Cu-MOF were recorded using UV–visible spectroscopy (Figure 3b). The strong absorption peaks of Cu-MOF are primarily located below 450 nm and above 620 nm, while the weak absorption peaks in the 450–620 nm range are attributed to the *d-d* transitions of Cu^2+^.^35^ A distinct exciton absorption peak for MAPbBr_3_-QDs appears at 532 nm, primarily attributed to the intraband transition of Pb^2+^ ions from the 6s to the 6p orbitals.^36^ Compared to the Cu-MOF, the overall absorption peaks of the MAPbBr_3_-QDs@Cu-MOF exhibit a blue shift of approximately 10 nm toward the lower wavelength region, which is mainly due to the change in color from green to yellow-green after the loading of the MAPbBr_3_-QDs into the Cu-MOF. Based on the UV–vis spectra, the bandgap energies of Cu-MOF, MAPbBr_3_-QDs, and MAPbBr_3_-QDs@Cu-MOF were determined using the Kubelka–Munk function (Figure 3c), yielding calculated values of 1.92, 2.17, and 2.09 eV, respectively (the solid-state UV–vis diffuse reflectance spectra and corresponding optical bandgap plots of the other materials are shown in Figure S12). The fluorescence emission peak of MAPbBr_3_-QDs@Cu-MOF (520 nm) exhibits a certain degree of blue shift compared to MAPbBr_3_-QDs (527 nm) (Figure S13). This suggests that the encapsulation of MAPbBr_3_-QDs in Cu-MOF can effectively control the size of the QDs. This phenomenon is primarily attributed to the influence of quantum effects, which lead to changes in the electronic energy levels, resulting in a shorter emission wavelength.

X-ray photoelectron spectroscopy (XPS) was employed to further investigate the composition and elemental valence states. In Figure S14, MAPbBr_3_-QDs@Cu-MOF exhibits not only the characteristic peaks of Cu-MOF but also the characteristic peaks of Pb and Br from MAPbBr_3_-QDs. The high-resolution XPS C 1s (284.6 eV) was used as a reference, and the high-resolution XPS spectra of other elements were analyzed (Figure S15a). The high-resolution XPS analysis of Cu 2p in Figure 3d reveals that in Cu-MOF, the binding energies of the main peaks are 935.19 and 955.20 eV, corresponding to Cu 2p3/2 and Cu 2p1/2, respectively, while the peaks at 940.64, 944.14, and 963.00 eV are satellite peaks. The valence state of Cu is +2.^37^ In MAPbBr_3_-QDs@Cu-MOF, the binding energies of Cu 2p3/2 and Cu 2p1/2 shift to lower binding energies by 0.81 and 0.96 eV, respectively, compared to Cu-MOF, with binding energies of 934.38 and 954.24 eV. Two additional peaks at 932.18 and 952.19 eV correspond to the binding energies of Cu^+^, indicating an increased electron density around some Cu^2+^ sites. In MAPbBr_3_-QDs@Cu-MOF, the binding energies of Pb 4f7/2 and Pb 4f5/2 are 138.56 and 143.34 eV, respectively (Figure 3e). Compared to the binding energies of Pb 4f7/2 (138.69 eV) and Pb 4f5/2 (143.55 eV) in MAPbBr_3_-QDs, these values shift to lower binding energies by 0.13 and 0.21 eV, respectively. The high-resolution XPS spectrum of Br 3d in MAPbBr_3_-QDs@Cu-MOF (68.29 eV) shifts to a lower binding energy by 0.12 eV compared to that of MAPbBr_3_-QDs (68.37 eV) (Figure 3f).^38^ The N 1s binding energy peaks of MAPbBr_3_-QDs, Cu-MOF, and MAPbBr_3_-QDs@Cu-MOF are located at 401.41, 399.72, and 400.31 eV, respectively (Figure S15b). The N 1s peak of the MAPbBr_3_-QDs@Cu-MOF shifts toward a lower binding energy compared to MAPbBr_3_-QDs, and toward a higher binding energy compared to Cu-MOF. This indicates that an electron transfer occurred from Cu-MOF to MAPbBr_3_-QDs.

### Third-order NLO properties of PeQDs@Cu-MOF

Highly transparent and soluble poly­(methyl methacrylate) (PMMA) was used as a substrate for film casting to investigate the third-order NLO properties of MAPbBr_3_-QDs@Cu-MOF. Linear absorption of the materials was recorded using UV–vis diffuse reflectance spectroscopy (Figure S16). After the materials were uniformly encapsulated in a PMMA substrate to form hybrid films with a dopant content of 0.5 wt %, no obvious light absorption was observed at 532 nm, ensuring that the exhibited properties are nonlinear.^39^ Figure S17 shows the SEM image of the hybrid film surface, further confirming that the hybrid film is dense, uniform, and free of cracks. Based on the SEM images showing a cross-section view of the hybrid film, the thicknesses of the Cu-MOF, MAPbBr_3_-QDs, and MAPbBr_3_-QDs@Cu-MOF hybrid films are approximately 0.4 mm. The elemental distribution images indicate that various elements are uniformly distributed within the materials, suggesting that Cu-MOF, MAPbBr_3_-QDs, and MAPbBr_3_-QDs@Cu-MOF are evenly dispersed in the PMMA substrate. The experimental results indicate that pure PMMA does not exhibit third-order NLO phenomena (Figure S18) and confirm that the NLO response of the hybrid film originates from the doping materials.

The strong quantum confinement effect and unique band structure in PeQDs are the fundamental reasons for the third-order NLO properties. Quantum confinement enhances the spatial overlap of electron and hole wave functions, thereby increasing the exciton binding energy. Meanwhile, the strong interaction between exciton–exciton and exciton-carrier enables PeQDs to exhibit NLA and NLR responses at high light intensities.^40–42^ The unique SBU structure (trinuclear Cu cluster SBU1 and tetranuclear Cu cluster SBU2) and the coordination environment (the d-orbital vacancy of the central metal Cu^2+^ forms a coordination bond with the electrons of the ligand L) in Cu-MOF, generates a strong charge transfer (metal–ligand charge transfer). Additionally, the porous structure and a highly ordered framework of Cu-MOF help regulate the spatial distribution of electrons and holes, providing favorable conditions for electron separation and migration of electrons and holes, which is conducive to the generation of NLO responses.^43,44^ Encapsulating bromide perovskites with small organic cations in Cu-MOF forms PeQDs with a size of 1.8 nm (typically ranging in size from a few nanometers to several tens of nanometers). The formation of these PeQDs nanocrystals effectively controls the spatial distribution of electrons and holes (enhancing quantum confinement effects), which influences their electronic structure and NLO properties.^45,46^ In addition, the internal charge transfer between the PeQDs and the Cu-MOF can generate spatially separated and long-lived electrons and holes, which may cause an increased distortion of the spatial electronic cloud when high-intensity laser light passes through the medium, thereby regulating the energy and phase of the laser, and producing superior third-order NLO performance.^47^

As shown in Figure 4a, under the femtosecond laser pulse conditions at a wavelength of 900 nm, the normalized transmission gradually increases as Cu-MOF moves along the *z*-axis. When Cu-MOF is positioned at the focal point *Z* = 0, the normalized transmittance reaches the maximum value of 1.18, and the experimental curve exhibits a peak, demonstrating saturable absorption (SA) behavior. In contrast, the NLA curve of MAPbBr_3_-QDs shows a valley, indicating reverse saturable absorption (RSA) behavior, with a third-order NLA coefficient (*β*_eff_) of 20 × 10^–12^ m/W. At the *Z* = 0 position, the minimum normalized transmission (*T*_min_) is 0.84. Subsequently, MAPbBr_3_-QDs-X (X = 5.7, 9.1, 17.6, 35.4, and 38.9 wt % ABBr_3_-QDs) with varying contents were loaded into the pores of Cu-MOF to investigate the third-order NLO properties of the PeQDs@Cu-MOF. As the concentration of MAPbBr_3_-QDs in Cu-MOF increases, the RSA behavior of MAPbBr_3_-QDs@Cu-MOF also intensifies. When the loading amount of MAPbBr_3_-QDs reaches 35.4 wt %, the RSA signal of MAPbBr_3_-QDs@Cu-MOF reaches the maximum, with a *β*_*eff*_ value of 120 × 10^–12^ m/W. However, when the content of MAPbBr_3_-QDs increases to 38.9 wt %, the *β*_*eff*_ value decreases to 50 × 10^–12^ m/W. The overload of MAPbBr_3_-QDs may destroy the Cu-MOF skeleton structure, thus reducing the NLA performance of MAPbBr_3_-QDs@Cu-MOF. When the wavelengths of the pulsed laser are 532 and 1064 nm, all materials exhibit the same NLO behavior as observed at a wavelength of 900 nm, but the intensities of NLA and NLR are different (Figures 4e–f and S19). This is primarily due to the difference in photon energy at different wavelengths, which results in varying interactions between the light waves and the electron clouds of materials, thereby causing changes in the intensity of the third-order NLO signal. The third-order NLA and NLR data for all materials are presented in Tables S1–S5.

**Figure 4:**
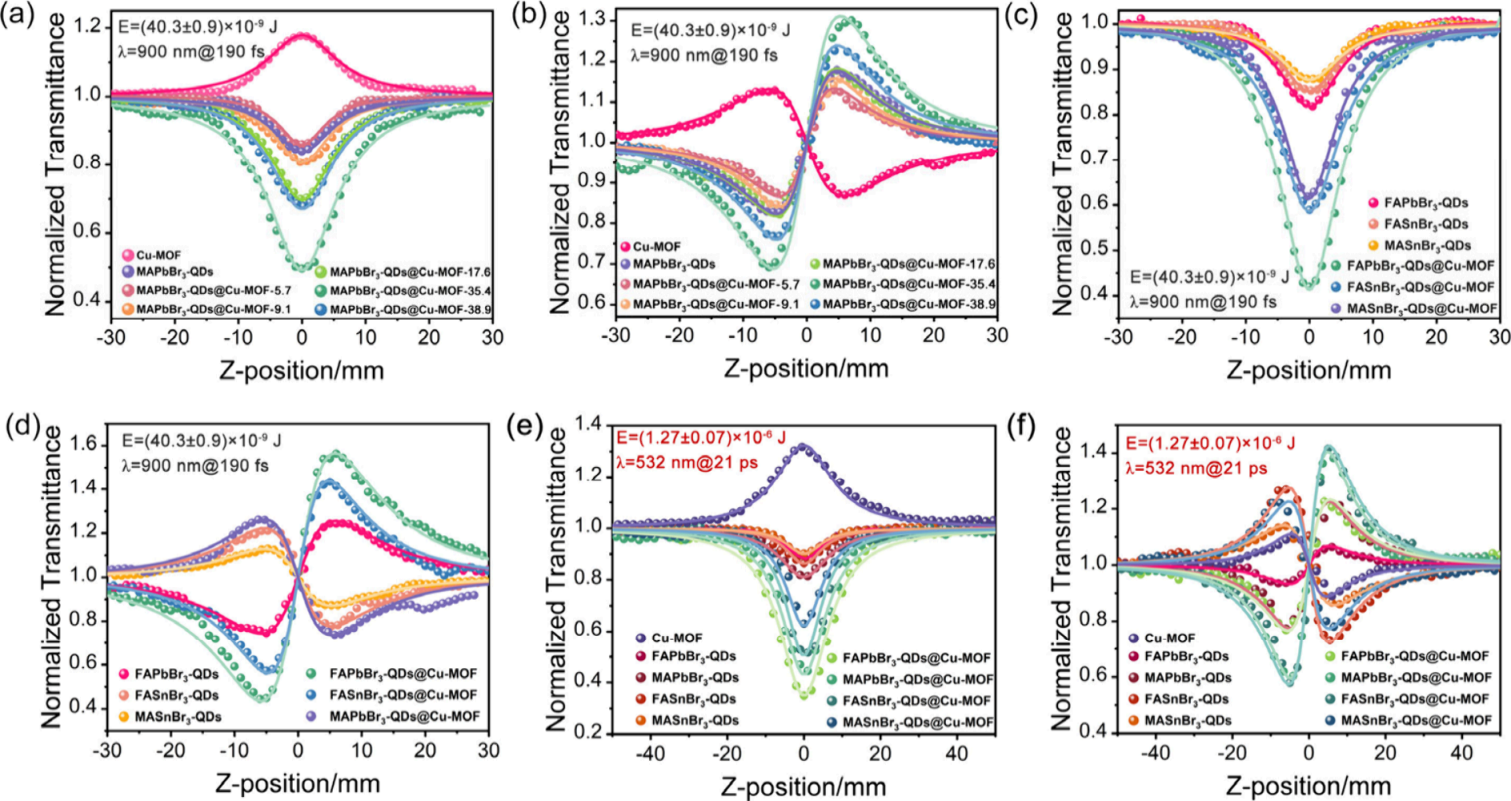
(a) NLA and (b) NLR results of Cu-MOF, MAPbBr_3_-QDs, and MAPbBr_3_-QDs@Cu-MOF-X at a pulsed wavelength of 900 nm; (c) NLA and (d) NLR results of FAPbBr_3_-QDs, MASnBr_3_-QDs, FASnBr_3_-QDs, FAPbBr_3_-QDs@Cu-MOF, MASnBr_3_-QDs@Cu-MOF and FASnBr_3_-QDs@Cu-MOF at a pulsed wavelength of 900 nm; (e) NLA and (f) NLR results of Cu-MOF, MAPbBr_3_-QDs, FAPbBr_3_-QDs, MASnBr_3_-QDs, FASnBr_3_-QDs, MAPbBr_3_-QDs@Cu-MOF, FAPbBr_3_-QDs@Cu-MOF, MASnBr_3_-QDs@Cu-MOF and FASnBr_3_-QDs@Cu-MOF at a pulsed wavelength of 532 nm. The measured and fit results are shown by data points and solid lines, respectively.

By spatially confining MAPbBr_3_-QDs within the cage-like cavities of the Cu-MOF, quantum-sized MAPbBr_3_-QDs composite materials are obtained, restricting the exciton motion of the MAPbBr_3_-QDs within the Cu-MOF (Exciton motion refers to the process of electron–hole pair migration within the matrix of PeQDs@Cu-MOF.).^48^ Consequently, the recombination of free charge carriers within the MAPbBr_3_-QDs is governed by the fine structure of the excitons. The splitting of the exciton fine structure is primarily caused by the exchange interactions between short-range and long-range electron–hole pairs, as well as the contributions of the crystal field introduced by the external Cu-MOF.^49^ In the MAPbBr_3_-QDs@Cu-MOF, the content of MAPbBr_3_-QDs can modulate the degree of enhancement of the RSA behavior. As the number of PeQDs increases, the intensity of RSA behavior also strengthens. This suggests that the quantum confinement effect enhances the spatial overlap between the electron and hole wave functions, thereby progressively increasing the interaction. The changes in the NLA signal of MAPbBr_3_-QDs@Cu-MOF, relative to the SA signal of Cu-MOF, are attributed to the incorporation of MAPbBr_3_-QDs. This incorporation leads to a more intense interaction between the electron–hole pairs within the material, significantly enhancing the generation and migration of photoexcited carriers, which enables MAPbBr_3_-QDs@Cu-MOF to exhibit RSA behavior.^50^ Compared to the RSA signal of MAPbBr_3_-QDs, the RSA signal of MAPbBr_3_-QDs@Cu-MOF is significantly enhanced. This enhancement is primarily attributed to the rapid diffusion of excitons and charge carriers, as well as the fast interface charge transfer between MAPbBr_3_-QDs and Cu-MOF. This process occurs on a picosecond (ps) time scale, facilitates effective exciton dissociation, generates photocurrents within the medium, promotes spatial charge rearrangement, and significantly enhances the third-order NLO properties of MAPbBr_3_-QDs.

Under the action of 900 nm pulsed laser, the third-order NLR behavior of Cu-MOF is characterized by self-defocusing, with a refractive index coefficient (*n*_2_) value of −90 × 10^–19^ m^2^/W. In contrast, MAPbBr_3_-QDs exhibit self-focusing refractive signals with an *n*_2_ value of 80 × 10^–19^ m^2^/W. As MAPbBr_3_-QDs are loaded into Cu-MOF to form MAPbBr_3_-QDs@Cu-MOF, the normalized transmittance exhibits a “valley–peak” pattern (Figure 4b). At the optimal loading amount, MAPbBr_3_-QDs@Cu-MOF exhibits the maximum self-focusing signal, with an *n*_2_ value of 158 × 10^–19^ m^2^/W.

The MAPbBr_3_-QDs@Cu-MOF exhibits self-focusing NLR behavior. The MAPbBr_3_-QDs@Cu-MOF medium can be considered a focusing lens, with the focal length changing in response to variations in laser intensity. When the MAPbBr_3_-QDs@Cu-MOF moves in front of the beam waist (*Z* < 0), the laser beam defocuses, reducing the laser energy passing through the aperture. Conversely, when MAPbBr_3_-QDs@Cu-MOF moves beyond the beam waist (*Z* > 0), the actual focus shifts further away, allowing more laser energy to pass through the small aperture and reach the detector.^26^ Compared to the self-defocusing behavior of Cu-MOF, the change in the NLR behavior of MAPbBr_3_-QDs@Cu-MOF arises from the presence of MAPbBr_3_-QDs, which provides an additional pathway for carrier transport, resulting in greater distortion of the spatial electronic cloud. This process modulates the phase of the laser beam within the medium, ultimately leading to a change in the third-order NLR response. The enhanced self-focusing effect in MAPbBr_3_-QDs@Cu-MOF, relative to MAPbBr_3_-QDs, is primarily attributed to two factors. First, when MAPbBr_3_-QDs is incorporated as QDs within Cu-MOF, the large potential barrier confines the carriers within the PeQDs, forming natural quantum wells.^51^ The quantum well structures, characterized by strong quantum confinement and dielectric constraints, can significantly enhance the self-focusing effect. Second, the interfacial charge transfer within the MAPbBr_3_-QDs@Cu-MOF occurs on a picosecond (ps) time scale, which matches the duration of the laser pulses. The spatial distortion of the electronic cloud caused by free carriers plays a dominant role in NLR behavior.^52^ As the content of MAPbBr_3_-QDs in Cu-MOF increases, the spatial coupling interaction gradually strengthens, leading to an enhancement of the NLR signal. The self-focusing signal reaches the maximum when the content of MAPbBr_3_-QDs increases to 35.4 wt %.

### The Effect of A/B-site Cations on the Third-Order NLO Properties of ABBr_3_-QDs@Cu-MOF

Due to their rich chemical composition, diverse structures, and advantages such as long carrier diffusion lengths, high oscillation strengths, and exciton binding energies, halide perovskites are being explored as promising NLO applications.^53^ The structural diversity of perovskites allows for the manipulation of the spatial dimensions by adjusting chemical composition or external conditions, which in turn influences their third-order nonlinear polarizability. Our investigation into the effect of cation composition in both the organic and inorganic dielectric layers of PeQDs provides deeper insights into how cation composition impacts their third-order NLO properties.

By tuning the A/B-cations within PeQDs, cation-dependent third-order NLO responses were achieved. At 900 nm wavelength laser irradiation, MAPbBr_3_-QDs, FAPbBr_3_-QDs, MASnBr_3_-QDs, and FASnBr_3_-QDs exhibit RSA characteristics, with normalized transmittances of 0.84, 0.82, 0.88, and 0.85, respectively, and corresponding the *β*_eff_ value are 20 × 10^–12^ m/W, 22 × 10^–12^ m/W, 13 × 10^–12^ m/W, and 18 × 10^–12^ m/W. After encapsulating these QDs in Cu-MOF, the MAPbBr_3_-QDs@Cu-MOF, FAPbBr_3_-QDs@Cu-MOF, MASnBr_3_-QDs@Cu-MOF, and FASnBr_3_-QDs@Cu-MOF exhibit enhanced RSA characteristics, with *β*_eff_ values of 120 × 10^–12^ m/W, 140 × 10^–12^ m/W, 57 × 10^–12^ m/W, and 72 × 10^–12^ m/W, respectively (Figure 4a and c). Compared to PeQDs, the *β*_eff_ value increased by a maximum of 6.36 times.

The A-site cations in ABBr_3_ occupy relatively large lattice positions in the PeQDs structure. The cations with different sizes and charges can lead to variations in the lattice dimensions of the PeQDs, thereby affecting the band gap structure of the material.^54–56^ When the larger FA^+^ cation replaces the smaller MA^+^, the larger size and stronger polarity of FA^+^ may facilitate the effective separation of electrons and holes, thereby increasing the *β*_eff_ of materials. In addition, the larger FA^+^ cation can also cause the Pb–Br bond length in the PeQDs to increase from 2.923 Å to 2.998 Å. A longer Pb–Br bond has weaker bond strength, which makes it easier for free charge carriers to migrate under the influence of an external optical field, thus enhancing the NLA response of materials.

The size and electronegativity of the B-site cation in ABBr_3_ determine the coupling strength between the electrons and lattice vibrations (phonons) in PeQDs. Sn-based perovskites are isostructural compounds of Pb-based perovskites. Although Sn-based perovskite materials possess a narrower optical bandgap, higher charge carrier mobility, and lower output binding energy, making them suitable for use as light-harvesting materials,^57^ the larger atomic radius of Pb^2+^ and stronger lattice distortion effects may facilitate stronger electronic delocalization, resulting in a greater electronic cloud oscillation intensity under an external optical field and enhanced the NLA performance. In addition, Pb^2+^ facilitates more stable incorporation into the perovskite lattice, reducing the formation of lattice defects and vacancies, thereby maintaining a higher carrier lifetime and lower carrier recombination under optical excitation, which further enhances the NLA performance. According to third-order NLO results, the NLA signal intensity of APbBr_3_-QDs@Cu-MOF is higher than that of ASnBr_3_-QDs@Cu-MOF.

The materials with RSA characteristics are promising candidates for optical limiter (OL) devices. A good optical limiting device exhibits a high transmittance at low input fluence while significantly restricting laser output power at high input fluence. We conducted OL tests on ABBr_3_-QDs@Cu-MOF at a pulse wavelength of 532 nm. In the relationship between normalized transmittance and input fluence (Figure S20), the normalized transmittance gradually decreases as the input fluence increases. The RSA properties of MAPbBr_3_-QDs@Cu-MOF are superior to those of MAPbBr_3_-QDs, indicating that the OL performance is also enhanced. The OL start threshold (*Fs*) for MAPbBr_3_-QDs@Cu-MOF is 19.2 × 10^–2^ J/cm^2^. As the input fluence increases, the normalized transmittance begins to decrease and reaches the optical limiting threshold (*Fol*) (the optical limiting threshold is defined as the input fluence corresponding to a normalized transmittance reduction to 0.5) at 39.5 × 10^–2^ J/cm^2^. It can be observed that the FAPbBr_3_-QDs@Cu-MOF exhibits the optimal OL performance, with *Fs* and *Fol* values of 8.05× 10^–2^ J/cm^2^ and 28.5 × 10^–2^ J/cm^2^, respectively. Figure S21 shows that the cation composition in PeQDs can be precisely tuned to modulate the OL properties of ABBr_3_-QDs@Cu-MOFs. This provides a feasible approach for the research and development of new OL materials.

When the pulse wavelength is 900 nm, the *n*_2_ values for MAPbBr_3_-QDs, FAPbBr_3_-QDs, MASnBr_3_-QDs, and FASnBr_3_-QDs are 80 × 10^–19^ m^2^/W, 123 × 10^–19^ m^2^/W, −59 × 10^–19^ m^2^/W, and −105 × 10^–19^ m^2^/W, respectively. While the *n*_*2*_ values of MAPbBr_3_-QDs@Cu-MOF, FAPbBr_3_-QDs@Cu-MOF, MASnBr_3_-QDs@Cu-MOF, and FASnBr_3_-QDs@Cu-MOF increase to 158 × 10^–19^ m^2^/W, 310 × 10^–19^ m^2^/W, −142 × 10^–19^ m^2^/W, and 210 × 10^–19^ m^2^/W, respectively. The NLR effects of PeQDs@Cu-MOF are significantly enhanced compared to ABBr_3_-QDs. Notably, the *n*_2_ values of MAPbBr_3_-QDs@Cu-MOF, FAPbBr_3_-QDs@Cu-MOF, and MASnBr_3_-QDs@Cu-MOF show a marked increase, indicating a positive enhancement in the nonlinear polarization of the composites. However, the NLR behavior of FASnBr_3_-QDs@Cu-MOF changes, indicating an opposite trend in nonlinear polarization (Figure 4b and d).

In the ABBr_3_-QDs@Cu-MOF composite material, the size of A/B-site cations and their charge delocalization range have a significant impact on the NLR properties of the material. Changes in the size of the A-site cations directly affect the lattice structure and bandgap of the PeQDs, while the B-site cations further modulate the NLR behavior by adjusting the electron distribution and local field effects.^58,59^ Larger A-site cations (FA^+^) and B-site cations (Pb^2+^), due to their larger ionic radii and stronger charge delocalization effects, can lead to more spatially dispersed electron distributions within the crystal, thereby enhancing the material’s polarization response. This electronic dispersion effect governs the material’s self-focusing behavior.^60–62^ When the A-site and B-site cations are substituted with smaller cations (such as MA^+^, and Sn^2+^), the reduced ionic radius leads to a decrease in the extent of electron delocalization, resulting in a self-defocusing behavior.

We observed that the NLR signal intensity of PeQDs@Cu-MOF is higher than that of PeQDs (e.g., MAPbBr_3_-QDs, FAPbBr_3_-QDs, and MASnBr_3_-QDs). After embedding FASnBr_3_-QDs into the Cu-MOF cavity, the NLR behavior undergoes a change. This phenomenon can be attributed to the synergistic interaction between the FASnBr_3_-QDs and Cu-MOF. In FASnBr_3_-QDs@Cu-MOF, the ionic radius of FA^+^ is larger than that of MA^+^, and the electronic system is more delocalized. The interfacial effect between Cu-MOF and FASnBr_3_-QDs may lead to the rapid reorientation of photogenerated carriers, the formation of larger polarons, and an increase in the activation energy for ion migration.^63^ The different ionic radii and electronegativity of Pb^2+^ and Sn^2+^ determine the coupling strength between electrons and lattice vibrations (phonons) in PeQDs, influencing the band structure and electron transport pathways of PeQDs. When Cu-MOF interacts with FASnBr_3_-QDs, the higher carrier mobility and lower output binding energy of FASnBr_3_-QDs may cause the phase of the wavefront at the focal point to be more easily altered under the influence of the laser field.^64^ This may explain the changes in the NLR behavior of FASnBr_3_-QDs and FASnBr_3_-QDs@Cu-MOF.

We have summarized the NLO properties of materials under femtosecond pulsed laser reported in recent years and conducted a comparative analysis. We have compared the NLO properties of various materials under femtosecond pulsed laser irradiation in recent years. The NLO response is not only significantly influenced by the material state (such as solution concentration or film thickness) and laser parameters (such as pulse width, energy and wavelength), but also closely related to the electronic structure of the material type (organic, inorganic and complexes, etc.). Therefore, there are significant differences in the NLO performance of different materials. According to the data in the Table S6 and Figure S22, the NLA and NLR coefficients of the composite materials are at a medium to high level compared to other materials, demonstrating outstanding nonlinear optical performance.

### Study of Carrier Dynamics in MAPbBr_3_-QDs@Cu-MOF

Using density functional theory (DFT) calculations, the frontier molecular orbitals of MAPbBr_3_-QDs@Cu-MOF, MAPbBr_3_-QDs, and Cu-MOF were investigated (Figure 5a), revealing the charge transfer characteristics of the different materials. In the HOMO and LUMO energy levels of MAPbBr_3_-QDs@Cu-MOF, the charge is predominantly distributed on the SBU1 of Cu-MOF and on MAPbBr_3_-QDs, indicating the presence of a charge transfer pathway between MAPbBr_3_-QDs and Cu-MOF, with intermolecular charge transfer from Cu-MOF to MAPbBr_3_-QDs. In MAPbBr_3_-QDs, the HOMO is mainly localized on the [PbBr_6_]^4–^ structure, while the LUMO is located on the MA^+^ cation, suggesting that [PbBr_6_]^4–^ acts as an electron donor and MA^+^ as an electron acceptor. For Cu-MOF, the charge transfer process occurs between the ligands and cluster nodes. The band structures of MAPbBr_3_-QDs@Cu-MOF, MAPbBr_3_-QDs, and Cu-MOF were calculated using DFT. The results indicate that the valence band maximum (VBM) and conduction band minimum (CBM) of MAPbBr_3_-QDs@Cu-MOF and Cu-MOF are 0.87 and 2.35 eV, 0.9 and 2.19 eV, respectively, with Fermi levels located within the VB, and the band gaps (Eg) are 1.48 and 1.29 eV, respectively (Figures 5b and S23). In the band structure of MAPbBr_3_-QDs (Figure 5c), both the CBM and VBM are located at the R-point, indicating that MAPbBr_3_-QDs is a direct band gap semiconductor. Density of states (DOS) analysis further reveals the influence of various elemental orbitals on the band structure of the composite material. As shown in Figure 5d, the VBM of Cu-MOF is mainly composed of O 2p, C 2p, and N 2p orbitals, while the CBM is primarily derived from C 2p and O 2p orbitals. For MAPbBr_3_-QDs, the CBM is mainly determined by Br 4p, and VBM is mainly determined by Pb 6s orbitals, suggesting that variations in the cations are significantly important for the band gap. In the MAPbBr_3_-QDs@Cu-MOF, the Br 4p, O 2p, and C 2p orbitals play a dominant role in the VBM and CBM. The considerable overlap between the Br 4p and O 2p orbitals suggests a strong interaction between Cu-MOF and MAPbBr_3_-QDs. The varying contributions of elemental orbitals to the band gap across different materials further lead to changes in the band structure, enabling effective modulation of the band gap.^65–67^

**Figure 5:**
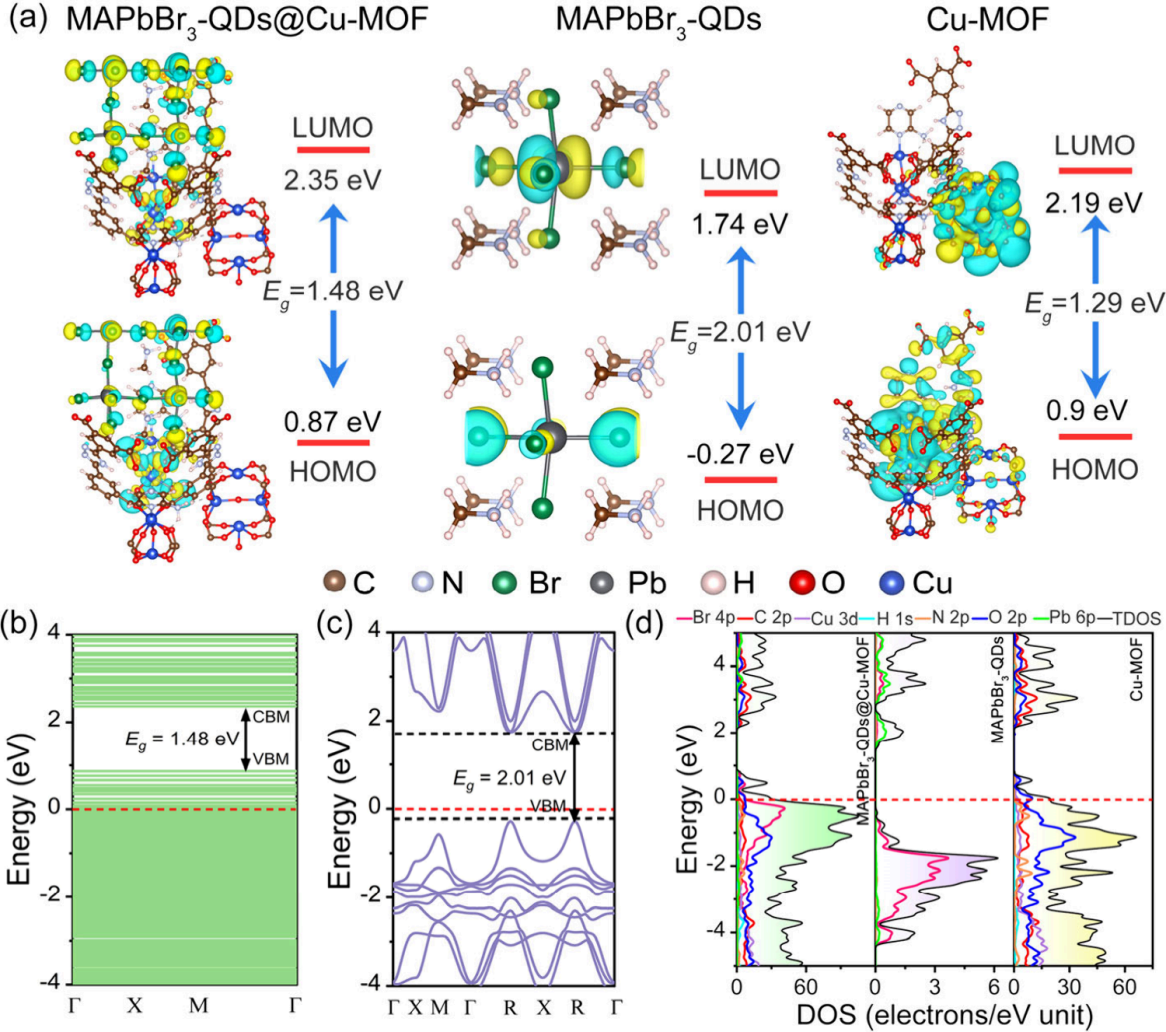
(a) Electron orbitals of HOMO and LUMO of MAPbBr_3_-QDs@Cu-MOF, MAPbBr_3_-QDs, and Cu-MOF; band structure of (b) MAPbBr_3_-QDs@Cu-MOF and (c) MAPbBr_3_-QDs; (d) total and partial DOS of MAPbBr_3_-QDs@Cu-MOF, MAPbBr_3_-QDs, and Cu-MOF.

To investigate the RSA process of MAPbBr_3_-QDs encapsulated in cage-like Cu-MOF, we employed fs-TAS with a 350 nm pump light and a pulse width of 100 fs, over a wavelength range of 440 to 700 nm, to explore the carrier dynamics of MAPbBr_3_-QDs@Cu-MOF, MAPbBr_3_-QDs and Cu-MOF on femtosecond to picosecond time scales. The two-dimensional pseudocolor maps and TAS of MAPbBr_3_-QDs and Cu-MOF are shown in Figures 6a-b and S24a-b, respectively. It can be observed that MAPbBr_3_-QDs exhibit a negative absorption band in the range of 480–526 nm, a characteristic bleach peak at 512 nm, and a broad absorption band around 550 nm.^68,69^ Cu-MOF shows a wide-range bleach peak below 554 nm and an excited-state absorption (ESA) signal above approximately 583 nm. Figure 6c presents the fast and slow recovery monitoring of MAPbBr_3_-QDs at 510 and 532 nm. The fast recovery time (τ_1_) is less than 1 ps, which is attributed to exciton trapping and results in the formation of self-trapped excitons.^70^ The slower recovery time (τ_2_) at 532 nm is longer compared to that at 510 nm, indicating a longer relaxation time for hot carriers, which may impact the extraction of carriers. This could explain the weaker RSA signal observed for MAPbBr_3_-QDs. The longer GSB recovery times at 532 nm for Cu-MOF (*τ*_1_ = 5.55 ps and *τ*_2_ = 369 ps) suggest that energy transfer processes are present within the Cu-MOF structure, leading to the dissipation of excited states (Figure S24c). In the TAS of the composite material MAPbBr_3_-QDs@Cu-MOF (Figure 6d–e), clear ESA peaks appear in the 480–650 nm range. As the delay time increases, the ESA signal gradually intensifies and begins to decay at 155 ps. The carrier dynamics delay curve indicates that the longer τ_1_ in MAPbBr_3_-QDs@Cu-MOF may represent the transfer of excitation energy from Cu-MOF to MAPbBr_3_-QDs, with electrons extending their relaxation time through interactions with other carriers. The smaller *τ*_*2*_ value suggests that electron–hole pairs can be efficiently transferred and trapped, further supporting the presence of charge transfer processes between Cu-MOF and MAPbBr_3_-QDs (Figure 6f).^71,72^

**Figure 6:**
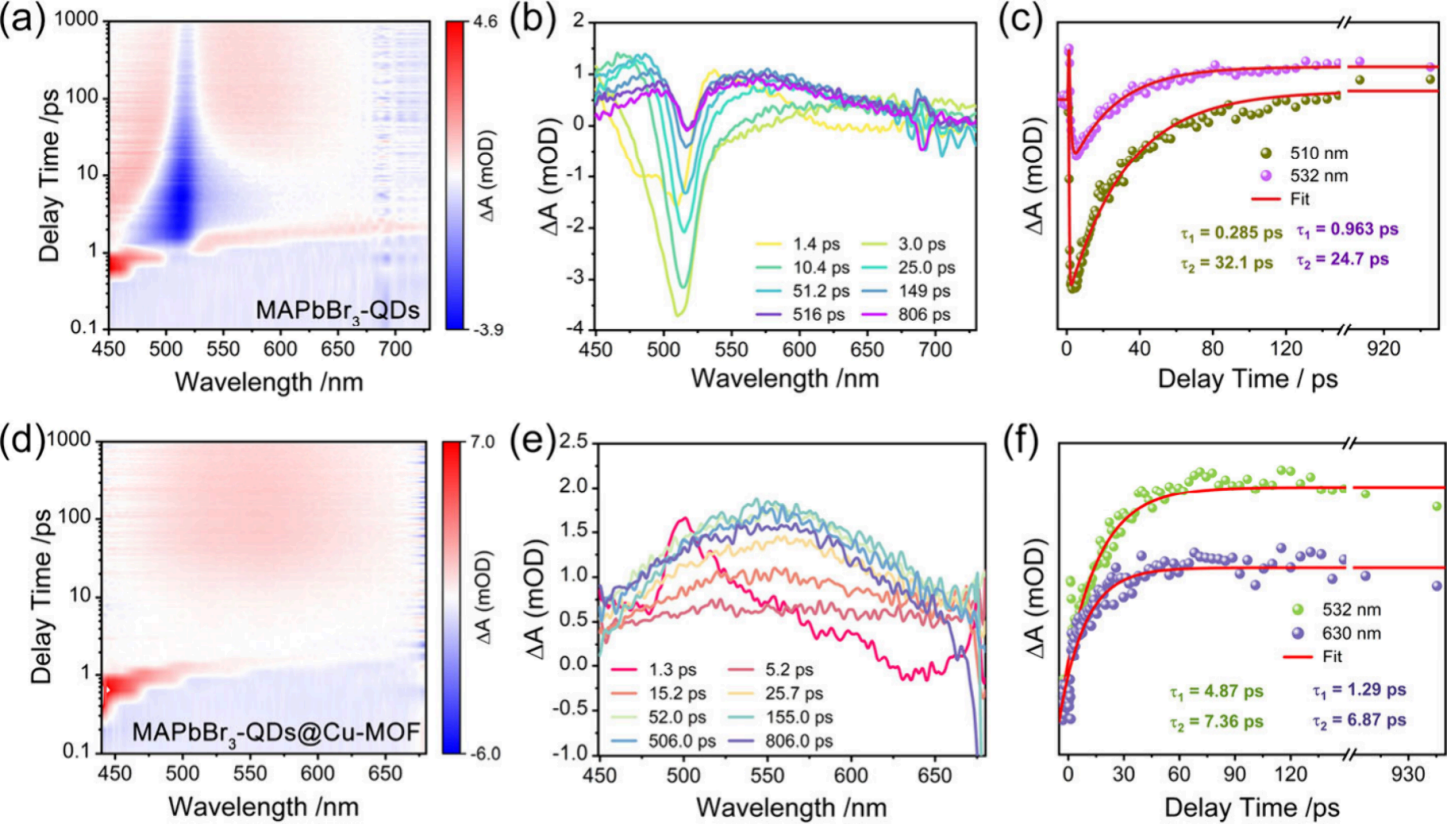
Two-dimensional pseudocolor TAS maps of (a) MAPbBr_3_-QDs and (d) MAPbBr_3_-QDs@Cu-MOF; TAS of (b) MAPbBr_3_-QDs and (e) MAPbBr_3_-QDs@Cu-MOF on the time (1.3 ps–806 ps) scales upon excitation at 350 nm; time-resolved TAS of (c) MAPbBr_3_-QDs and (f) MAPbBr_3_-QDs@Cu-MOF at different wavelengths.

To determine the optical nonlinearity order of the RSA of MAPbBr_3_-QDs and MAPbBr_3_-QDs@Cu-MOF, the relationship between the normalized transmittance change (1 – *T*_0_, i.e., the amplitude of the valley in the Z-scan curve) and the laser pulse energy (*E*_pulse_) was investigated. In principle, the slope n of the ln­(*E*_pulse_) versus ln­(1 – *T*_0_) curve corresponds to the nominal (*n*+1)-photon absorption.^73,74^ Under 532 nm laser excitation (Figure S25), the slope of MAPbBr_3_-QDs and MAPbBr_3_-QDs@Cu-MOF are 0.32 and 0.76, respectively, indicating that the NLO process was a two-photon absorption (TPA). According to the pump–probe spectra, when the delay time is zero, the normalized transmittance of MAPbBr_3_-QDs@Cu-MOF drops rapidly to 0.81 and then starts to rise quickly, forming a distinct “sharp valley” phenomenon. Subsequently, a “turning point” occurs at approximately 130 ps, and the normalized transmittance does not return to the linear absorption level (i.e., the normalized transmittance of 1). This indicates that the carrier dynamics process in MAPbBr_3_-QDs@Cu-MOF is influenced by both TPA and ESA. The pump–probe spectrum of MAPbBr_3_-QDs shows that the normalized transmittance drops sharply to 0.90 at zero delay time and then recovers rapidly, eventually returning to the linear absorption, presenting a clear “sharp valley” feature. This suggests that MAPbBr_3_-QDs mainly undergo a TPA process (Figure S26).^75^

## Experimental Details

### Preparation of ABBr_3_-QDs@Cu-MOF-X (X = 5.7, 9.1, 17.6, 35.4, and 38.9 wt % ABBr_3_-QDs)

0.022 mmol (0.050 g) of Cu-MOF was immersed in 2 mL of BBr_2_ (0.1, 0.2, 0.4, 1.0, or 1.2 mmol) anhydrous DMF solution and stirred for 2 h. Afterward, the BBr_2_@Cu-MOF was washed with anhydrous DMF to remove any residual BBr_2_ on the surface. Subsequently, the BBr_2_@Cu-MOF was immersed in 2 mL of anhydrous DMF containing of ABr (0.1, 0.2, 0.4, 1.0, or 1.2 mmol) and stirred at room temperature for 2 h. The solid sample was then washed multiple times with anhydrous DMF to remove any remaining BBr_2_ and ABr on the surface, ensuring both BBr_2_ and ABr in the cavities of the Cu-MOF. Finally, an excess amount of toluene was added to form ABBr_3_-QDs@Cu-MOF-X. The entire process was carried out under a N_2_ atmosphere for protection. When X = 35.4 wt %, ABBr_3_-QDs@Cu-MOF-35.4 is abbreviated as ABBr_3_-QDs@Cu-MOF.

### Z-Scan Measurements

The laser source, provided by an optical parametric amplifier (Light Conversion ORPHEUS) pumped by a femtosecond fiber laser (Light Conversion Pharos–SP), emits wavelengths of 900 and 1064 nm with a repetition rate of 20 Hz and a pulse duration of 190 ps (FWHM). During the Z-scan measurement, the sample moves along the *z*-axis relative to the focus of *f* = 200 mm focal length lens. Effective third-order NLO absorption coefficient *β*_eff_ is determined and fitted using formulas [Disp-formula eq1] and 2:

1 $$T_{OA}=1+1/\beta_{eff}L_{eff}I_{0}\left(1/1+{\left(Z/Z_{0}\right)}^{2}\right)$$

2 $$L_{eff}=\left(1-\exp\left(-\alpha L\right)\right)/\alpha$$

The *T*_OA_ is a function of the *Z* position of the sample (relative to the focal point at *Z* = 0), *L*_eff_ is the effective length of the sample, α is the linear absorption coefficient, *L* is the sample thickness, *I*_0_ is the peak light intensity, *Z*_0_ = kω_0_^2^/2 is the Rayleigh range, *k* is the wave vector, ω_0_ is the waist radius at the focal position.

The nonlinear refractive index *n*_*2*_ under closed aperture is confirmed by formulas [Disp-formula eq3] and 4:

3 $$T\left(z,\Delta \Phi_{0}\left(t\right)\right)=1-\frac{4\Delta \Phi_{0}\left(t\right)z}{z_{0}\left(1+\frac{z^{2}}{z_{0}^{2}}\right)\left(9+\frac{z^{2}}{z_{0}^{2}}\right)}$$

4 $$\Delta \Phi_{0}\left(t\right)=kn_{2}I_{0}\left(t\right)L_{eff}$$

ΔΦ_0_(*t*) is the phase change of the wavefront at the focal point (*z* = 0), and *n*_2_ is the third-order nonlinear refractive index.

### TAS Measurement

The transient absorption spectra (TAS) were recorded on a commercial pump–probe system (Helios EOS, Ultrafast Systems LLC) in combination with a femtosecond laser system (Astrella, Coherent). Laser pulses (∼800 nm center wavelength, < 100 fs duration, 1 kHz repetition rate, ∼ 7 mJ/pulse) were generated by a Ti: sapphire-based regenerative amplifier laser system. The laser pulses were split to generate pump and probe beams. The pump pulses at 350 nm was delivered by an optical parametric amplifier (OPA) that was excited by a portion of the 800 nm laser pulses. The pump pulse energy in each measurement was ∼ 10 μJ/cm^2^ at the sample cell. For femtosecond TA (100 fs -7 ns delay region) measurement, the probe and reference beams were generated by focusing the 800 nm beam (split from the amplifier with a tiny portion) onto a CaF_2_ crystal to generate white-light continuum pulses (350–700 nm). The pump–probe delay was controlled by an optical delay line. For the nanosecond TA (1 ns -400 μs delay region) measurement, the pump beam was generated in the same way as in the femtosecond TA experiment described above. The probe and reference beams were provided by an additional supercontinuum laser (370–900 nm, ∼ 0.5 ns duration, 2 kHz repetition rate). The pump–probe delay time was electronically controlled in the nanosecond TA measurement. In the femtosecond and nanosecond TA spectrometer, the intensities of the probe and reference beams were detected by a pair of linear array detectors to obtain the TA signal. Global and target analyses were performed with the Glotaran software. The femtosecond and nanosecond TA data were combined for the convenience of global analysis.

## Conclusion

In summary, this study utilizes the unique cavity confinement effect to encapsulate ABBr_3_-QDs within the cage-like cavity of a Cu-MOF, resulting in uniformly dispersed ABBr_3_-QDs. By reducing the perovskite size to the quantum scale, the interaction with the ABBr_3_-QDs and Cu-MOF interface reconfigures the electron cloud density and optimizes carrier transport pathways, leading to significant enhancement or modification of the third-order NLO properties. Furthermore, variations in the A/B-site cations of ABBr_3_-QDs@Cu-MOF enable precise tuning of the materials’ third-order NLO characteristics. The band structure and carrier dynamics of ABBr_3_-QDs@Cu-MOF are analyzed through DFT calculations and fs-TAS, revealing the intrinsic reasons for regulating the third-order NLO properties. The idea offers new insights for the development of advanced third-order NLO materials.

## Supplementary Material



## Notes

Additional experimental details and supporting charts: PXRD, crystal structure diagrams, UV–vis spectra, fluorescence spectra, N_2_ adsorption isotherms, TEM, SEM, EDS, XPS, Z-scan figures, fs-TAS, and tables of all materials’ NLO data (PDF)
