# Supplementary material for: Cage Confinement Strategy in Perovskite-QDs@MOF for Boosting the Third-Order Nonlinear Optical Performance
Source: ACS Cent Sci. 2025 Jul 23;11(9):1627–39. doi: 10.1021/acscentsci.5c00863 (PMC12464757; doi:10.1021/acscentsci.5c00863)
Supplement: Supplementary file 1 [file oc5c00863_si_001.pdf]

Supporting Information

Cage Confinement Strategy in Perovskite-QDs@MOF for Boosting the Third-Order Nonlinear Optical Performance

Yupei Sun, Kangshuai Geng, Jing Huang, Yi Wei, Hongwei Hou*

College of Chemistry, Zhengzhou University, Zhengzhou, Henan 450001, China

E-mail: houhongw@zzu.edu.cn

List of Contents

Experimental Section S1

Preparation of Cu-MOF S1

Preparation of $ABBr_3$-QDs..S2

Preparation of $ABBr_3$-QDs@Cu-MOF-X (X = 5.7 wt%, 9.1 wt%, 17.6 wt%, 35.4 wt%, and 38.9 wt% $ABBr_3$-QDs)..S2

Preparation of PMMA film S3

Preparation of Cu-MOF/PMMA film S3

Preparation of $ABBr_3$-QDs/PMMA film..S3

Preparation of $ABBr_3$-QDs@Cu-MOF-X/PMMA film S3

Characterizations..S3

Z-Scan Measurements..S4

TAS measurement S7

Computational details S8

Supplementary Figures and Tables S9

Figure S1. The kinetic curves of Co-Cu metal ion exchange (The illustration shows photographs of the crystal during the exchange process). S9

Figure S2. The asymmetric unit, SBU1, SBU2, the shape and size of the cavity, and 3D framework structure of Cu-MOF. S9

Figure S3. The crystal structure and molecular size of $ABBr_3$-QDs. S10

Figure S4. (a) Image of Cu-MOF crystal; (b-c) Images of $MAPbBr_3$-QDs@Cu-MOF; (d) Cross-sectional image of $MAPbBr_3$-QDs@Cu-MOF. S10

Figure S5. The powder XRD patterns of $ABBr_3$-QDs@Cu-MOF, Cu-MOF, and $ABBr_3$-QDs. S11

Figure S6. EDS mapping and line scanning of $MAPbBr_3$-QDs@Cu-MOF..S12

Figure S7. EDS mapping of the cross-section of $MAPbBr_3$-QDs@Cu-MOF. S12

Figure S8. SEM images of $MAPbBr_3$-QDs at (a) 200 nm and (b) 100 nm scales. S13

Figure S9. TEM image of $MAPbBr_3$-QDs@Cu-MOF at a 200 nm scale. S13

Figure S10. (a) N_2 adsorption-desorption isotherms of Cu-MOF, $MAPbBr_3$-QDs, and

$MAPbBr_3$-QDs@Cu-MOF; (b) Pore size distributions are calculated from the adsorption branch of isotherms based on the NLDFT model. S13

Figure S11. FT-IR of $MAPbBr_3$-QDs@Cu-MOF, $MAPbBr_3$-QDs, and Cu-MOF. 1 is Cu-MOF, 2 is $MAPbBr_3$-QDs, and 3 is $MAPbBr_3$-QDs@Cu-MOF. S14

Figure S12. (a) Solid-state UV-Vis absorption spectra and (b) the optical band gap of $FAPbBr_3$-QDs, $MASnBr_3$-QDs, and $FASnBr_3$-QDs; (c) Solid-state UV-Vis absorption spectra and (d) the optical band gap of $FAPbBr_3$-QDs@Cu-MOF, $MASnBr_3$-QDs@Cu-MOF, and $FASnBr_3$-QDs@Cu-MOF. S15

Figure S13. PL spectrum of $MAPbBr_3$-QDs@Cu-MOF, $MAPbBr_3$-QDs and Cu-MOF. ..S15

Figure S14. XPS spectra of Cu-MOF, $MAPbBr_3$-QDs, and $MAPbBr_3$-QDs@Cu-MOF..S16

Figure S15. (a) High-resolution C 1s XPS spectra of Cu-MOF, $MAPbBr_3$-QDs, and $MAPbBr_3$-QDs@Cu-MOF; (b) High-resolution N 1s XPS spectra of Cu-MOF, $MAPbBr_3$-QDs, and $MAPbBr_3$-QDs@Cu-MOF. S16

Figure S16. Solid-state UV-Vis absorption spectra of PMMA, Cu-MOF, $MAPbBr_3$-QDs, and $MAPbBr_3$-QDs@Cu-MOF films. S17

Figure S17. Surface and cross-sectional SEM images of $MAPbBr_3$-QDs@Cu-MOF/PMMA, as well as element distribution maps of Cu, Pb, Br, C, O, N. S18

Figure S18. The NLA data and NLR data of pure PMMA film at a pulsed wavelength of 532 nm...S18

Figure S19. (a) NLA and (b) NLR results of Cu-MOF, $MAPbBr_3$-QDs, $FAPbBr_3$-QDs, $MASnBr_3$-QDs, $FASnBr_3$-QDs, $FAPbBr_3$-QDs@Cu-MOF, $MAPbBr_3$-QDs@Cu-MOF, $MASnBr_3$-QDs@Cu-MOF and $FASnBr_3$-QDs@Cu-MOF at a pulsed wavelength of 1064 nm...S18

Figure S20. (a) Normalized transmittance of the $MAPbBr_3$-QDs, $MAPbBr_3$-QDs@Cu-MOF, and $FAPbBr_3$-QDs@Cu-MOF at the beam waist is related to the input fluence; (b) Output fluence of the $MAPbBr_3$-QDs, $MAPbBr_3$-QDs@Cu-MOF, and $FAPbBr_3$-QDs@Cu-MOF at the beam waist is related to the input fluence. S19

Figure S21. (a) Normalized transmittance of the $MASnBr_3$-QDs@Cu-MOF and $FASnBr_3$-QDs@Cu-MOF at the beam waist is related to the input fluence; (b) Output fluence of the $MASnBr_3$-QDs@Cu-MOF and $FASnBr_3$-QDs@Cu-MOF at the beam waist is related to the

input fluence..S19

Figure S22. NLO properties of different materials under femtosecond pulsed laser........S20

Figure S23. Band structure of Cu-MOF. S20

Figure S24 . (a) Two-dimensional pseudocolor TAS maps of Cu-MOF; (b) TAS of Cu-MOF on the time (0.55 ps - 805 ps) scales upon excitation at 350 nm; (c) Time-resolved TAS of Cu-MOF at different wavelengths. S21

Figure S25. Plot of ln(1-T_0) versus ln(E_{pulse}) to determine the order of optical nonlinearity of (a) $MAPbBr_3$-QDs and (b) $MAPbBr_3$-QDs@Cu-MOF. S21

Figure S26. The transient absorption spectrum of (a) $MAPbBr_3$-QDs@Cu-MOF, (b) $MAPbBr_3$-QDs, and (c) Cu-MOF. S21

Table S1. Third-order NLO parameters of Cu-MOF, $MAPbBr_3$-QDs, $MAPbBr_3$-QDs@Cu-MOF-5.7, $MAPbBr_3$-QDs@Cu-MOF-9.1, $MAPbBr_3$-QDs@Cu-MOF-17.6, $MAPbBr_3$-QDs@Cu-MOF-35.4, and $MAPbBr_3$-QDs@Cu-MOF-38.9 under 532 nm laser irradiation. S24

Table S2. Third-order NLO parameters of Cu-MOF, $MAPbBr_3$-QDs, $FAPbBr_3$-QDs, $MASnBr_3$-QDs, $FASnBr_3$-QDs, $MAPbBr_3$-QDs@Cu-MOF, $FAPbBr_3$-QDs@Cu-MOF, $MASnBr_3$-QDs@Cu-MOF, and $FASnBr_3$-QDs@Cu-MOF under 532 nm laser irradiation. S25

Table S3. Third-order NLO parameters of Cu-MOF, $MAPbBr_3$-QDs, $MAPbBr_3$-QDs@Cu-MOF-5.7, $MAPbBr_3$-QDs@Cu-MOF-9.1, $MAPbBr_3$-QDs@Cu-MOF-17.6, $MAPbBr_3$-QDs@Cu-MOF-35.4, and $MAPbBr_3$-QDs@Cu-MOF-38.9 under 900 nm laser irradiation. S26

Table S4. Third-order NLO parameters of Cu-MOF, $MAPbBr_3$-QDs, $FAPbBr_3$-QDs, $MASnBr_3$-QDs, $FASnBr_3$-QDs, $MAPbBr_3$-QDs@Cu-MOF, $FAPbBr_3$-QDs@Cu-MOF, $MASnBr_3$-QDs@Cu-MOF, and $FASnBr_3$-QDs@Cu-MOF under 900 nm laser irradiation. S27

Table S5. Third-order NLO parameters of Cu-MOF, $MAPbBr_3$-QDs, $FAPbBr_3$-QDs, $MASnBr_3$-QDs, $FASnBr_3$-QDs, $MAPbBr_3$-QDs@Cu-MOF, $FAPbBr_3$-QDs@Cu-MOF, $MASnBr_3$-QDs@Cu-MOF, and $FASnBr_3$-QDs@Cu-MOF under 1064 nm laser irradiation. S28

Table S6. The NLA and NLR coefficients of different materials under femtosecond pulsed laser and the corresponding references. S29
References..S31

Experimental Section

Materials and reagents: All materials and reagents were obtained from commercial sources and have not been further purified.

Preparation of Cu-MOF: The Cu-MOF was synthesized following the established procedure described in the literature.[1] $CoCl_2·6H_2O$ (23.8 mg, 0.1 mmol), 5,5'-(1H-2,3,5-triazole-1,4-diyl)diisophthalic acid(H_4L, 19.8 mg, 0.05 mmol), and 2-Pyrazinamine (Pz-NH_2, 9.5 mg, 0.1 mmol) were dissolved in 5 mL of *N,N*-dimethylformamide (DMF), followed by adding three drops of concentrated nitric acid (The mass fraction was 68%). The mixture was then sealed in a 10 mL glass vial and kept at 100 °C for 48 h and naturally cooled down to room temperature. 1 mL of deionized water was added to the mixture and further kept at 100 °C for 12 h, followed by cooling naturally. The formed hexagonal-shaped purple crystals of Co-MOF were collected and washed three times with DMF. Through single crystal to single crystal center metal exchange, Co-MOF can be transformed into Cu-MOF. The specific experimental process involves soaking 1 equivalent of Co-MOF in a 0.1M solution of $Cu(NO_3)_2·6H_2O$ in acetonitrile and maintaining it at 80 °C for 6 hours. This process is repeated three times until the crystal color changes from purple to green and the solution no longer undergoes any changes.

The molecular formula in structures is usually determined based on the chemical stoichiometry of the unit cell, the asymmetric structural unit, or the smallest repeating unit. The determination of the stoichiometric coefficients in the Cu-MOF molecular formula is based on the asymmetric unit in the structure. In the Cu-MOF, due to high crystallographic symmetry, the L^{4-} had to be modeled with strict restraints and constraints, and the thermal vibration parameters of all the non-hydrogen atoms were constrained to be the same with isotropic model. In addition, the occupancy of the L^{4-} was artificially fixed at 0.25 considering the chemical and crystallographic reasonability. The -NH_2 on Pz-NH_2 was disordered over four crystallographically equal positions, whose geometry was restrained using "DFIX" and "DANG" commands. The atomic displacement parameters of the C and amino-N atoms of Pz-NH_2 were kept isotropic with fixed values. The occupancy of each O atom of the coordinated water molecules was determined based on the difference Fourier map. Hydrogen atoms of the H_2O were not added, but they were counted in the molecular formula. All the Cu cations in this structure were with 1 chemical occupancy. The asymmetric unit of the structure contains 1.75

Cu^{2+}, corresponding to +3.5 charges, 0.75 L^{4-} (-3 charges), 0.125 μ_3-O^{2-} (-0.25 charges) and 0.25 μ_2-OH^- (-0.25 charges), forming an electrically neutral framework. Squeeze was applied to treat the residual density in the cavity of the structure. Based on the results of Squeeze, the structure contains in total of three CH_3CN molecules. Therefore, the molecular formula of Cu-MOF is ($\{[Cu_{1.75}L_{0.75}(Pz\text{-}NH_2)_{0.125}(\mu_3\text{-}O)_{0.125}(\mu_2\text{-}OH)_{0.25}(H_2O)_{0.375}]\cdot3CH_3CN\}_n$, where L = 5,5'-(1H-2,3,5-triazole-1,4-diyl)diisophthalic acid).

To verify whether Co-MOF was completely transformed into Cu-MOF, we recorded the Co-Cu exchange process using an atomic absorption spectrometer (AAS) (AA7020 model) and a stereomicroscope (Leica S9i series). The parent Co-MOF is a purple hexagonal prism crystal. After three hours of central metal ion exchange, the crystal color changed from "green-brown-purple" from the edge to the center, indicating partial exchange of the Co-MOF crystal. According to the AAS analysis, the percentages of Co^{2+} and Cu^{2+} ions were calculated to be 32:68. After six hours, the crystal color completely turned green, and no characteristic atomic absorption peak of Co^{2+} was detected, indicating that Co^{2+} had been completely replaced by Cu^{2+}. The specific phenomenon is shown in Figure S1.

Preparation of $ABBr_3$-QDs: The 1.0 mmol of BBr_2 (B = Pb, Sn) and 1.0 mmol of ABr (A = MA (methylammonium), FA (formamidine)) were each dissolved in 2 ml of anhydrous DMF to form precursor solutions. After thoroughly mixing the BBr_2 and ABr solutions, toluene was added in excess to precipitate $ABBr_3$-QDs.

Preparation of $ABBr_3$-QDs@Cu-MOF-X (X = 5.7 wt%, 9.1 wt%, 17.6 wt%, 35.4 wt%, and 38.9 wt% $ABBr_3$-QDs): 0.022 mmol (0.050 g) of Cu-MOF was immersed in 2 mL of BBr_2 (0.1, 0.2, 0.4, 1.0, or 1.2 mmol) anhydrous DMF solution and stirred for 2 hours. Afterward, the BBr_2@Cu-MOF was washed with anhydrous DMF to remove any residual BBr_2 on the surface. Subsequently, the BBr_2@Cu-MOF was immersed in 2 mL of anhydrous DMF containing of ABr (0.1, 0.2, 0.4, 1.0, or 1.2 mmol) and stirred at room temperature for 2 hours. The solid sample was then washed multiple times with anhydrous DMF to remove any remaining BBr_2 and ABr on the surface, ensuring both BBr_2 and ABr in the cavities of the Cu-MOF. Finally, an excess amount of toluene was added to form $ABBr_3$-QDs@Cu-MOF-X. The entire process was carried out under a N_2 atmosphere for protection. When X = 35.4 wt%, $ABBr_3$-QDs@Cu-MOF-35.4 is abbreviated as $ABBr_3$-QDs@Cu-MOF.

Preparation of PMMA film: The polymethyl methacrylate (PMMA) with a mass of 1.194 g should be dissolved in 6 mL of anhydrous acetonitrile solvent to produce a colorless and transparent colloidal solution. The PMMA was uniformly coated onto a quartz glass plate using the solution casting technique. A pure PMMA film with an approximate thickness of 0.40 mm was obtained after the solvent evaporated.

Preparation of Cu-MOF/PMMA film: The PMMA with a mass of 1.194 g should be dissolved in 6 mL of anhydrous acetonitrile solvent to yield a colorless and transparent colloidal solution. The Cu-MOF with a dosage of 6 mg was subsequently dispersed in a PMMA gel-like liquid through ultrasonic treatment and stirred for 3 hours to form the Cu-MOF. The Cu-MOF was uniformly applied onto a quartz glass plate using the solution casting technique. Cu-MOF films with a mass fraction of 0.5 wt% and a thickness of approximately 0.40 mm were obtained after solvent evaporation.

Preparation of ABBr$_3$-QDs/PMMA film: The PMMA with a mass of 1.194 g should be dissolved in 6 mL of anhydrous acetonitrile solvent to yield a colorless and transparent colloidal solution. The ABBr$_3$-QDs with a dosage of 6 mg was subsequently dispersed in a PMMA gel-like liquid through ultrasonic treatment and stirred for 3 hours to form the ABBr$_3$-QDs. The ABBr$_3$-QDs was uniformly applied onto a quartz glass plate using the solution casting technique. ABBr$_3$-QDs films with a mass fraction of 0.5 wt% and a thickness of approximately 0.40 mm were obtained after solvent evaporation.

Preparation of ABBr$_3$-QDs@Cu-MOF-X/PMMA film: The PMMA with a mass of 1.194 g should be dissolved in 6 mL of anhydrous acetonitrile solvent to yield a colorless and transparent colloidal solution. The ABBr$_3$-QDs@Cu-MOF-X with a dosage of 6 mg was subsequently dispersed in a PMMA gel-like liquid through ultrasonic treatment and stirred for 3 hours to form the ABBr$_3$-QDs@Cu-MOF-X. The ABBr$_3$-QDs@Cu-MOF-X was uniformly applied onto a quartz glass plate using the solution casting technique. ABBr$_3$-QDs@Cu-MOF-X films with a mass fraction of 0.5 wt% and a thickness of approximately 0.40 mm were obtained after solvent evaporation.

Characterizations

Power X-ray diffraction (PXRD) patterns of all compounds were recorded on PANalytical X'Pert Pro diffractometer with Cu-Kα radiation (λ=1.54184 Å) in the air at room temperature (RT). UV-vis

diffuse reflectance spectra were measured using a JASCO-750 UV-visible spectrophotometer in the wavelength range of 200-800 nm, with $BaSO_4$ as a reference, and Fourier transform infrared (FT-IR) spectra were collected on a Bruker 27 tensor spectrophotometer in the 400-4000 cm^{-1} range. The X-ray photoelectron (XPS) measurements were carried out an using X-ray photoelectron spectrometer (Thermo Fischer, ESCALAB 250Xi Al kα-ray, USA). Transmission electron microscopy (TEM) images were obtained using An FEI TecNAIG2F20-S-Twin electron microscope operated at 300 KV. TEM specimens were prepared by depositing a small amount of the sample solution onto carbon-coated copper grids. The phase formation of the material was analyzed using Raman spectroscopy. All spectra were recorded at room temperature with a HORIBA LabRAM HR Evolution system equipped with a TE-cooled charge-coupled device (CCD) array detector. The samples were excited using a 633 nm HeNe laser with a power of 7.25 mW, and the laser beam was focused into a spot with a diameter of approximately 1 mm using a microscope objective lens (magnification: 100×). The material cross-sectional samples were observed using an aberration-corrected STEM microscope (Titan G2 60-300, Thermo Fisher, USA; equipped with a field emission gun) at an accelerating voltage of 300 kV. To minimize damage to the MHP framework during atomic-resolution imaging, the electron probe current was reduced to 5 pA. The probe convergence angle was 24.5 mrad, and the angle range of the HAADF detector was from 79.5 to 200 mrad. During STEM-HAADF image acquisition, the dwell time per pixel was set to 6 μs. The material was observed using a next-generation cryo-transmission electron microscope, Glacios (Thermo Fisher Scientific, equipped with an X-FEG electron gun), with an accelerating voltage range of 80 kV to 200 kV and a beam current of 1.2 nA. Data were collected using the EPU single-particle automation system, and images were acquired with the Falcon4 next-generation direct electron detector. The actual loading amounts were determined by atomic absorption spectrometry (AAS). In $MAPbBr_3$-QDs@Cu-MOF, the loading amounts of $MAPbBr_3$-QDs range from low to high at 5.7 wt%, 9.1 wt%, 17.6 wt%, 35.4 wt%, and 38.9 wt%, respectively. For $FAPbBr_3$-QDs@Cu-MOF, $MASnBr_3$-QDs@Cu-MOF, and $FASnBr_3$-QDs@Cu-MOF, the loading amounts of PeQDs are 34.5 wt%, 36.8 wt%, and 32.3 wt%, respectively.

Z-Scan Measurements

Nonlinear optics is a physical phenomenon related to the high-order polarization of media. When light is incident on a medium, under the influence of the amplitude of the external light field, the molecules, atoms, or charges in the medium will undergo overall or relative displacements, thereby

inducing a polarized electric field (represented by the physical quantity of polarization intensity). In the case of ordinary light, the polarization intensity is proportional to the amplitude of the external light field, and the polarizability is linear. However, when the incident light is a laser, the light intensity is several orders of magnitude higher than that of ordinary light. At this time, the polarization intensity can be expanded as a power series of the electric field, and the effect of higher-order terms needs to be considered. When the power exponent is 2 or 3, it corresponds to second-order or third-order nonlinear polarization. Nonlinear absorption (NLA) effect is a phenomenon that occurs when the electrons in the ground state of the medium absorb photon energy and undergo energy level transitions under the action of a laser, and the NLA coefficient of the medium changes with the light intensity. It mainly includes two-photon absorption, saturation absorption, and reverse saturation absorption, etc. The nonlinear refraction (NLR) effect is that when a Gaussian laser beam passes through the medium, the molecular orientation and electron cloud density change, resulting in an uneven refractive index distribution in the radial direction of the beam, forming a phenomenon similar to concave or convex lenses, thereby increasing or decreasing the beam size in the transverse direction, that is self-focusing or self-defocusing.

The third-order nonlinear optical (NLO) picosecond data of the film were recorded using the NLO-IZ Integrated instrument for picosecond Z-scan and optical limiting (Suzhou Micro-Nano Laser Photonics Technology Co., Ltd.). A Q-switched Nd: YAG laser emits a green laser with a wavelength of 532 nm as the light source, providing a repetition rate of 10 Hz and a pulse width of 21 ps (FWHM). The lens has a focal length of f = 400 mm, and the radius of the incident light spot is 3×10^{-3} m. The distance from the lens focal point to the plane of the aperture is D = 0.47 m. The beam waist size ranges from 46 to 49 μm. The pump light energy data is recorded by a detector (Rj-765a, Laser Probe). CuPcTs/DMSO (4×10^{-5} mol L^{-1}) is used as the standard correction optical path.

The laser source, provided by an optical parametric amplifier (Light Conversion ORPHEUS) pumped by a femtosecond fiber laser (Light Conversion Pharos−SP), emits wavelengths of 900 nm and 1064 nm with a repetition rate of 20 Hz and a pulse duration of 190 ps (FWHM). During the Z-scan measurement, the sample moves along the z-axis relative to the focus of f = 200 mm focal length lens.

During the measurement process, the sample is moved back and forth along the Z-axis (the direction of laser propagation) near the focus. The curves obtained from this system are referred to as

Z-scan curves. Each time the sample is moved to a different Z position, D1 and D2 record a set of data, resulting in two experimental curves: one is the "open-aperture (OA)" Z-scan curve, which represents third-order NLA data (D2/D1). Since D2 detects the total energy of the entire beam passing through the sample, it reflects the NLA at different positions within the sample. The other is the "closed-aperture (CA)" Z-scan curve, obtained by placing a small aperture A in front of the D2 detector, and representing third-order NLR data (D2/D1). It reflects the energy variation near the optical axis on the measurement plane, which is related to both NLR and NLA. For samples exhibiting both NLR and NLA, the experimental CA curve is influenced by the strength of the NLA. To isolate the contribution of NLR, the CA curve is often divided by the OA curve, which removes the absorption effects and yields a curve that is approximately due to pure refraction.

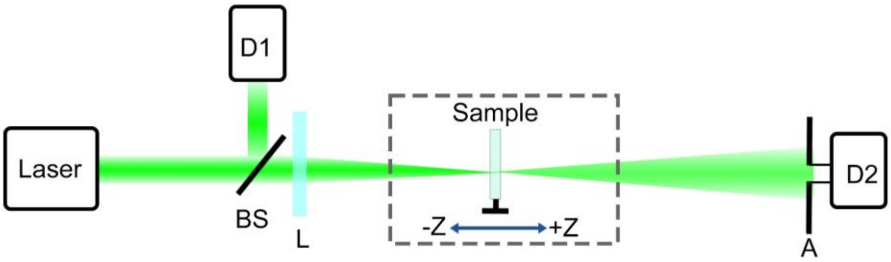

The Z-scan schematic diagram. BS is the beam splitter, L is the lens, and D1 and D2 are energy meters.

Effective third-order NLO absorption coefficient β_{eff} is determined and fitted using formulas (1) and (2):

$$T_{OA} = 1 + 1/\beta_{eff} L_{eff} I_0 (1/1 + (Z/Z_0)^2) \quad (1)$$

$$L_{eff} = {(1 - \exp(-\alpha L))}/{\alpha} \quad (2)$$

The T_{OA} is a function of the Z position of the sample (relative to the focal point at Z=0), L_{eff} is the effective length of the sample, α is the linear absorption coefficient, L is the sample thickness, I_0 is the peak light intensity, $Z_0=k\omega_0^2/2$ is the Rayleigh range, k is the wave vector, ω_0 is the waist radius at the focal position.

The nonlinear refractive index n_2 under closed aperture is confirmed by formulas (3) and (4):

$$T(z, \Delta\Phi_0(t)) = 1 - \frac{4\Delta\Phi_0(t) z}{z_0(1+\frac{z^2}{z_0^2})(9+\frac{z^2}{z_0^2})} \quad (3)$$

$$\Delta\Phi_0(t) = k n_2 I_0(t) L_{eff} \quad (4)$$

$\Delta\Phi_0(t)$ is the phase change of the wavefront at the focal point (z=0), and n_2 is the third-order

nonlinear refractive index. When the fitting curve shows a "peak valley" pattern, $n_2<0$, indicates the self-defocusing of the sample; When the fitting curve shows a "valley peak" pattern, $n_2>0$, it indicates the sample is self-focused; When the fitting curve presents a straight line, $n_2=0$, and the sample has no refraction phenomenon.

Source of experimental error: During the Z-scan measurements, fluctuations in the output power of the laser source may occur, which can affect the measurement results and introduce errors. Under irradiation of a 532 nm wavelength laser, the selected laser energy is 1.27×10^{-6} J, with an energy fluctuation range from 1.22×10^{-6} J to 1.34×10^{-6} J. Under irradiation of a 900 nm wavelength laser, the selected laser energy is 40.0×10^{-9} J, with an energy fluctuation range from 39.4×10^{-6} J to 40.5×10^{-6} J. Under irradiation of a 1064 nm wavelength laser, the selected laser energy is 37.0×10^{-9} J, with an energy fluctuation range from 36.4×10^{-6} J to 37.4×10^{-6} J. We choose to determine the light energy before each Z-scan measurement and record it as the incident light energy.

To eliminate the nonlinear response of PMMA, we have taken the following measures:

Background Correction: In our experiment, we used a blank PMMA sample for background measurements to evaluate the intrinsic nonlinear effects. Under the same conditions, we performed Z-scan tests on pure PMMA films (0.4 mm thick). The experimental results indicate that pure PMMA does not exhibit third-order NLO phenomena (Figure S14). By comparing the Z-scan results of the hybrid films with those of pure PMMA, we were able to eliminate the influence of PMMA and confirm that the NLO response of the hybrid film originates from the doping materials.

Improvement of the Measurement Procedure: To ensure that the measured results reflect only the properties of the material itself, we minimized the influence of the PMMA during the experimental process. For example, we reduced the laser energy (1.27 μJ at 532 nm wavelength) and conducted Z-scan measurements at longer wavelengths (900 nm and 1064 nm), with laser energies of 40.3 nJ and 36.95 nJ, respectively, to minimize the NLO response and linear absorption of PMMA. Moreover, we incorporated corrections for the response of PMMA during the data fitting procedure to accurately obtain the Z-scan data of the materials.

TAS measurement

The transient absorption spectra (TAS) were recorded on a commercial pump-probe system (Helios EOS, Ultrafast Systems LLC) in combination with a femtosecond laser system (Astrella, Coherent). Laser pulses (~800 nm center wavelength, < 100 fs duration, 1 kHz repetition rate, ~7 mJ/pulse) were

generated by a Ti: sapphire-based regenerative amplifier laser system. The laser pulses were split to generate pump and probe beams. The pump pulses at 350 nm was delivered by an optical parametric amplifier (OPA) that was excited by a portion of the 800 nm laser pulses. The pump pulse energy in each measurement was ~10 μJ/cm^2 at the sample cell. For femtosecond TA (100 fs - 7 ns delay region) measurement, the probe and reference beams were generated by focusing the 800 nm beam (split from the amplifier with a tiny portion) onto a CaF_2 crystal to generate white-light continuum pulses (350-700 nm). The pump-probe delay was controlled by an optical delay line. For the nanosecond TA (1 ns - 400 μs delay region) measurement, the pump beam was generated in the same way as in the femtosecond TA experiment described above. The probe and reference beams were provided by an additional supercontinuum laser (370-900 nm, ~0.5 ns duration, 2 kHz repetition rate). The pump-probe delay time was electronically controlled in the nanosecond TA measurement. In the femtosecond and nanosecond TA spectrometer, the intensities of the probe and reference beams were detected by a pair of linear array detectors to obtain the TA signal. Global and target analyses were performed with the Glotaran software. The femtosecond and nanosecond TA data were combined for the convenience of global analysis.

Computational details

All of the calculations are performed in the framework of the spin-polarized density functional theory with the projector augmented plane-wave method, as implemented in the Vienna ab initio simulation package (VASP).[2, 3] The generalized gradient approximation (GGA) proposed by Perdew, Burke, and Ernzerhof (PBE) is selected for the exchange-correlation potential.[4, 5] The long-range van der Waals interaction is described by the DFT-D3 approach.[6] The cut-off energy for plane wave is set to 480 eV. The energy criterion is set to 10^{-5} eV in iterative solution of the Kohn-Sham equation. All the structures are relaxed until the residual forces on the atoms have declined to less than 0.02 eV/Å. Data analysis and visualization are carried out with the help of VASPKIT[7] code and VESTA[8]. To avoid interlaminar interactions, a vacuum spacing of 20 Å is applied perpendicular to the slab.

Supplementary Figures and Tables

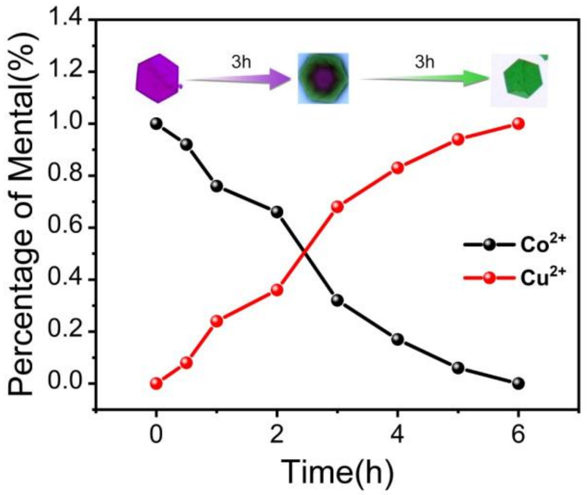

Figure S1. The kinetic curves of Co-Cu metal ion exchange (The illustration shows photographs of the crystal during the exchange process).

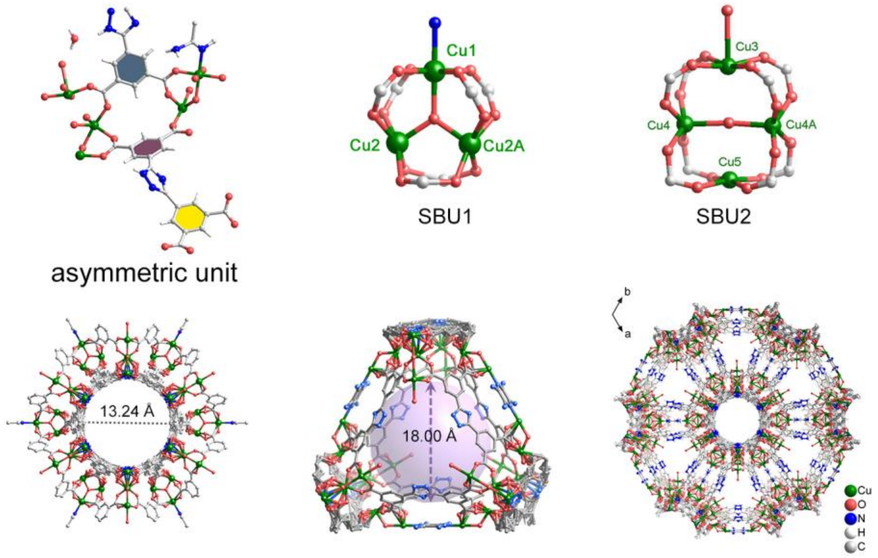

Figure S2. The asymmetric unit, SBU1, SBU2, the shape and size of the cavity, and 3D framework structure of Cu-MOF.

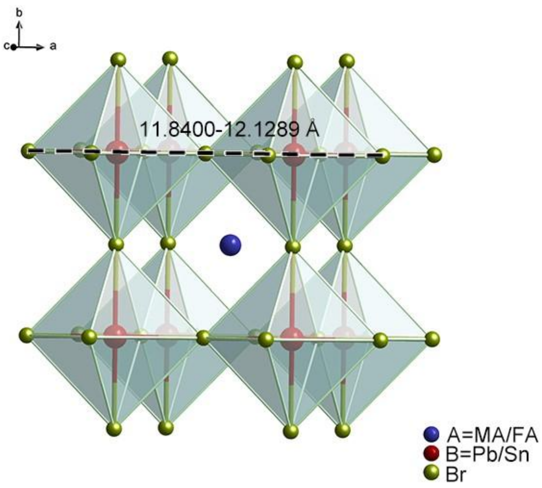

Figure S3. The crystal structure and molecular size of $ABBr_3$-QDs.

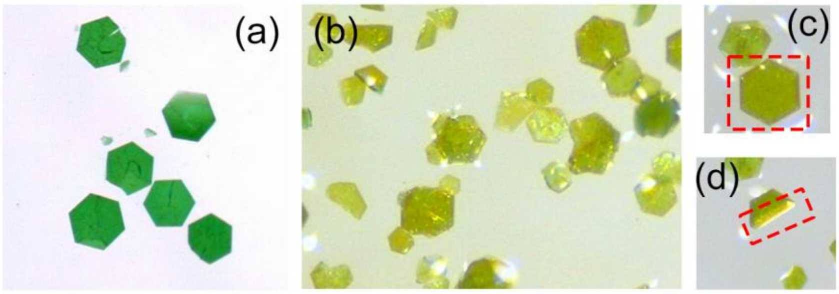

Figure S4. (a) Image of Cu-MOF crystal; (b-c) Images of $MAPbBr_3$-QDs@Cu-MOF; (d) Cross-sectional image of $MAPbBr_3$-QDs@Cu-MOF.

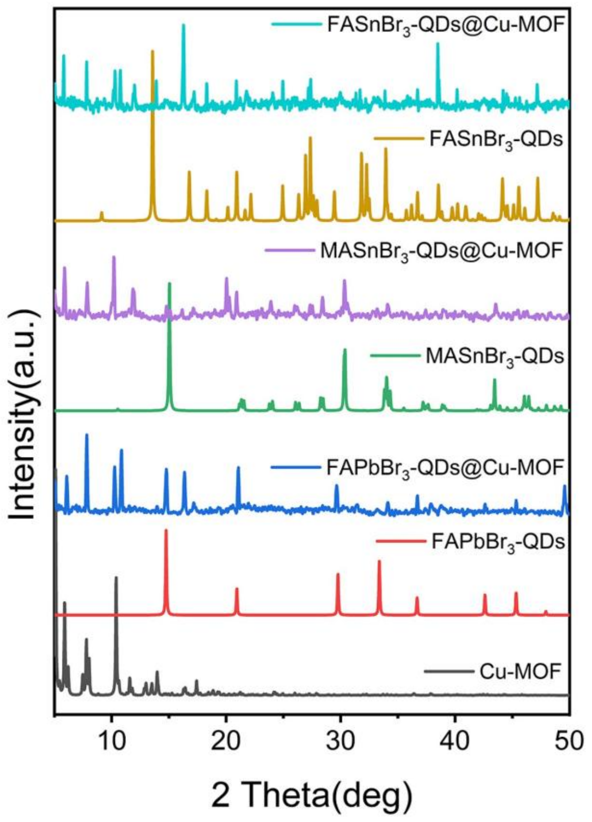

Figure S5. The powder XRD patterns of $ABBr_3$-QDs@Cu-MOF, Cu-MOF, and $ABBr_3$-QDs.

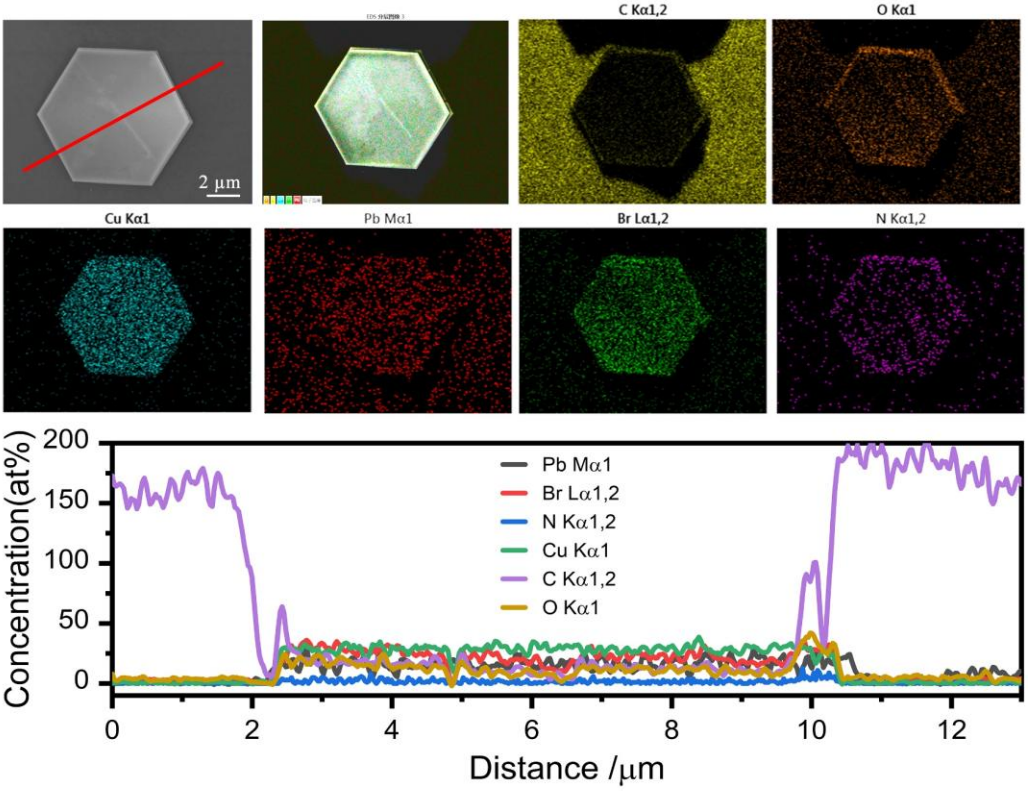

Figure S6. EDS mapping and line scanning of $MAPbBr_3$-QDs@Cu-MOF.

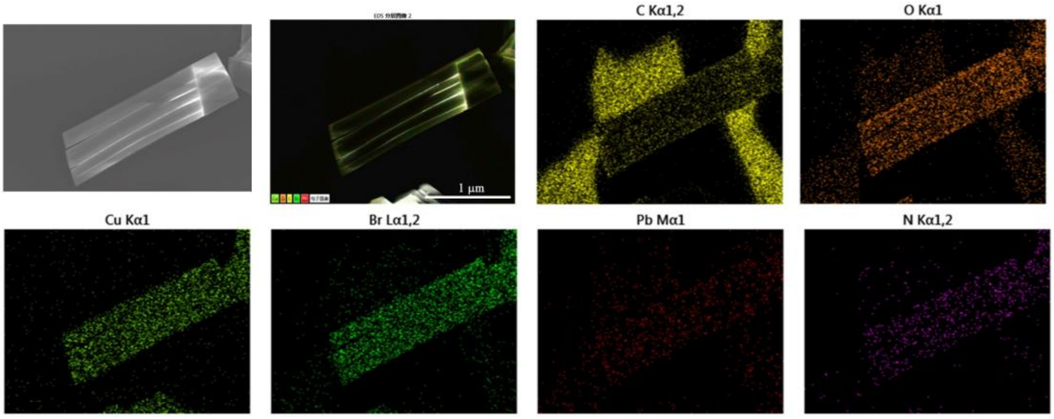

Figure S7. EDS mapping of the cross-section of $MAPbBr_3$-QDs@Cu-MOF.

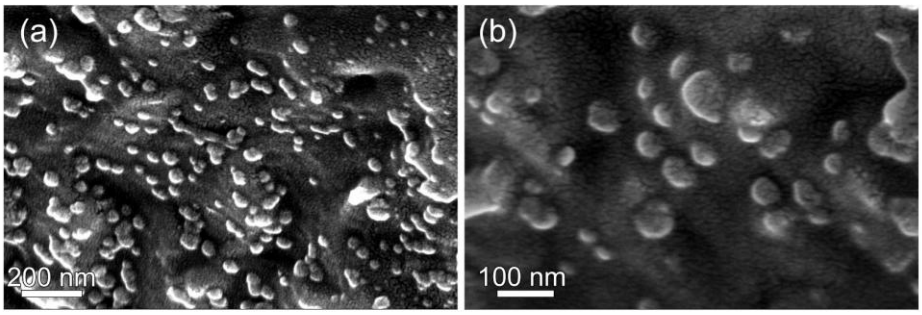

Figure S8. SEM images of $MAPbBr_3$-QDs at (a) 200 nm and (b) 100 nm scales.

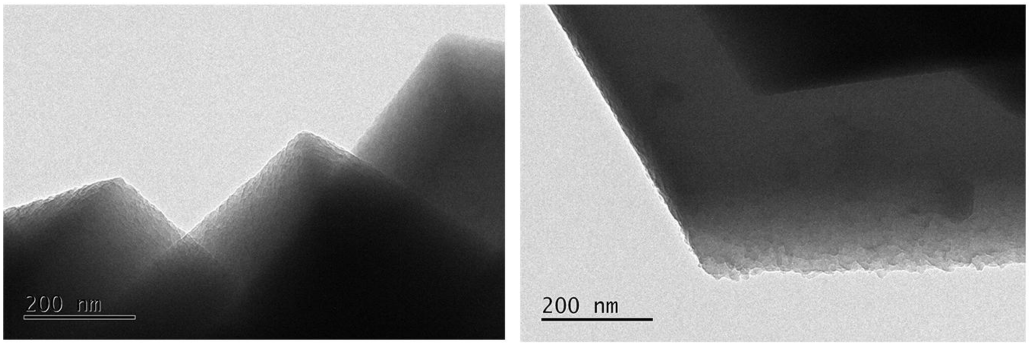

Figure S9. TEM image of $MAPbBr_3$-QDs@Cu-MOF at a 200 nm scale.

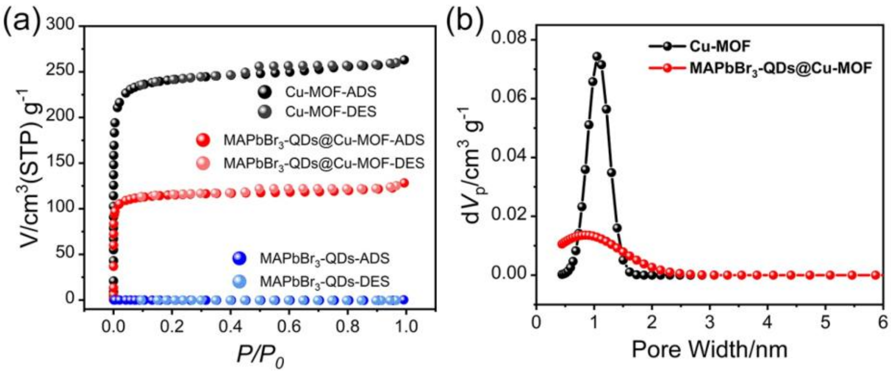

Figure S10. (a) N_2 adsorption-desorption isotherms of Cu-MOF, $MAPbBr_3$-QDs, and $MAPbBr_3$-QDs@Cu-MOF; (b) Pore size distributions are calculated from the adsorption branch of isotherms based on the NLDFT model.

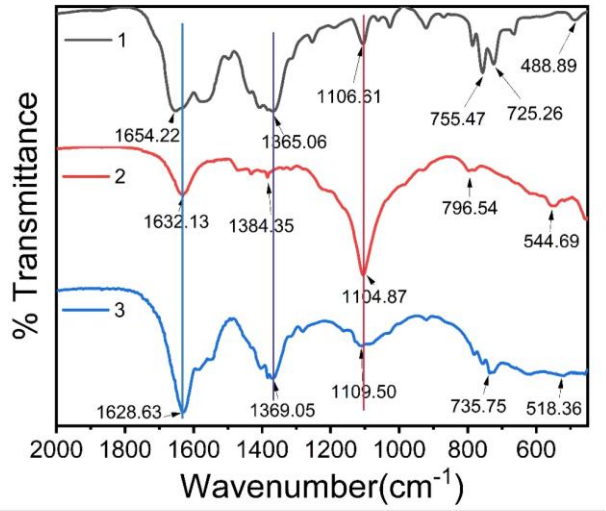

Figure S11. FT-IR of $MAPbBr_3$-QDs@Cu-MOF, $MAPbBr_3$-QDs, and Cu-MOF. 1 is Cu-MOF, 2 is $MAPbBr_3$-QDs, and 3 is $MAPbBr_3$-QDs@Cu-MOF.

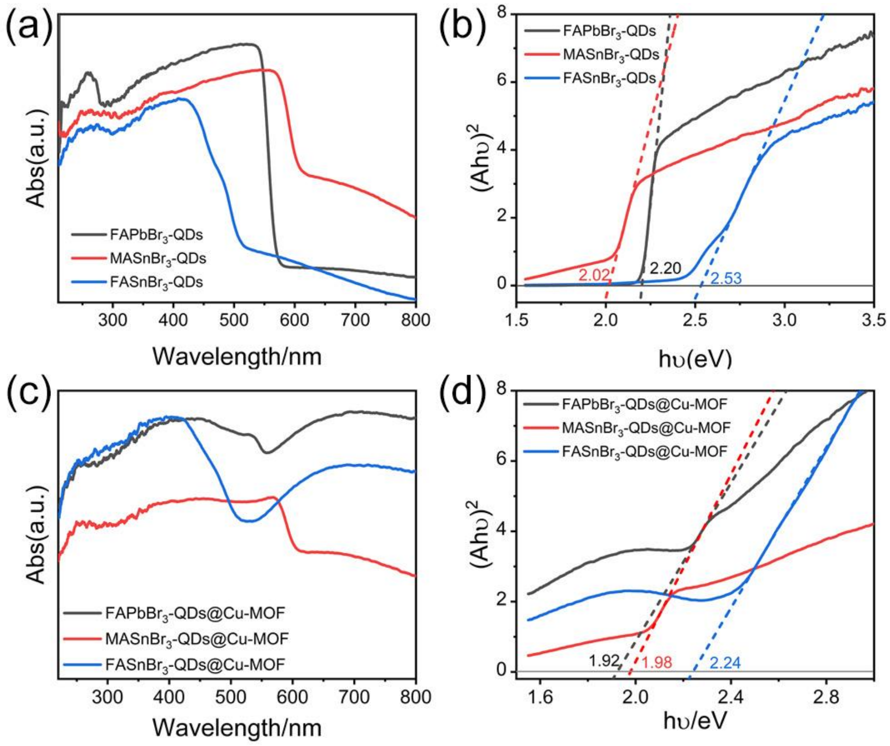

Figure S12. (a) Solid-state UV-Vis absorption spectra and (b) the optical band gap of $FAPbBr_3$-QDs, $MASnBr_3$-QDs, and $FASnBr_3$-QDs; (c) Solid-state UV-Vis absorption spectra and (d) the optical band gap of $FAPbBr_3$-QDs@Cu-MOF, $MASnBr_3$-QDs@Cu-MOF, and $FASnBr_3$-QDs@Cu-MOF.

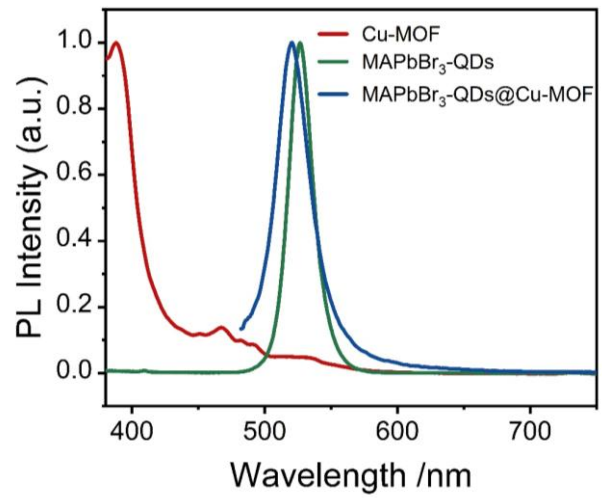

Figure S13. PL spectrum of $MAPbBr_3$-QDs@Cu-MOF, $MAPbBr_3$-QDs and Cu-MOF.

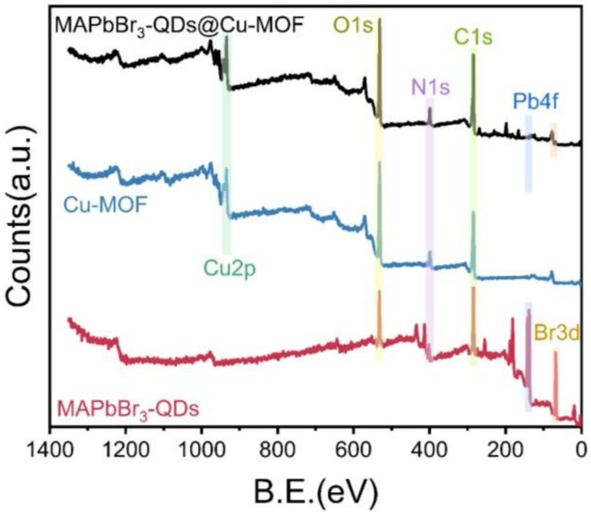

Figure S14. XPS spectra of Cu-MOF, $MAPbBr_3$-QDs, and $MAPbBr_3$-QDs@Cu-MOF.

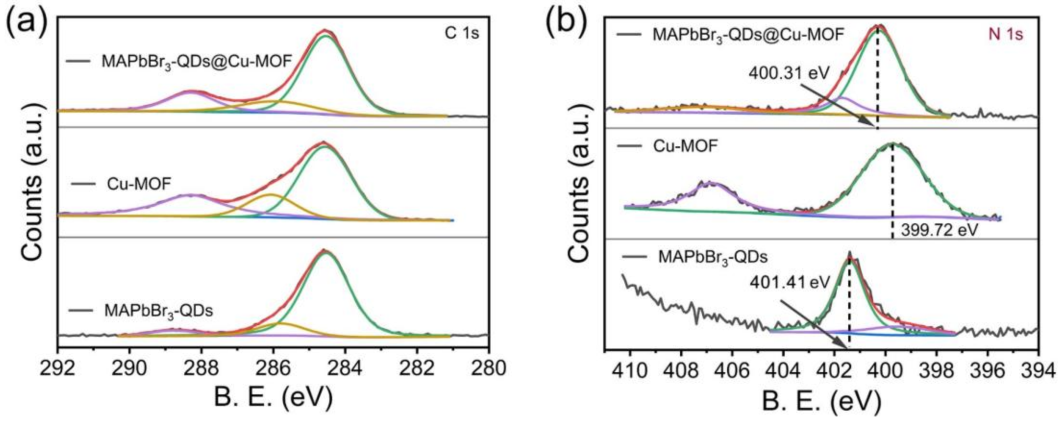

Figure S15. (a) High-resolution C 1s XPS spectra of Cu-MOF, $MAPbBr_3$-QDs, and $MAPbBr_3$-QDs@Cu-MOF; (b) High-resolution N 1s XPS spectra of Cu-MOF, $MAPbBr_3$-QDs, and $MAPbBr_3$-QDs@Cu-MOF.

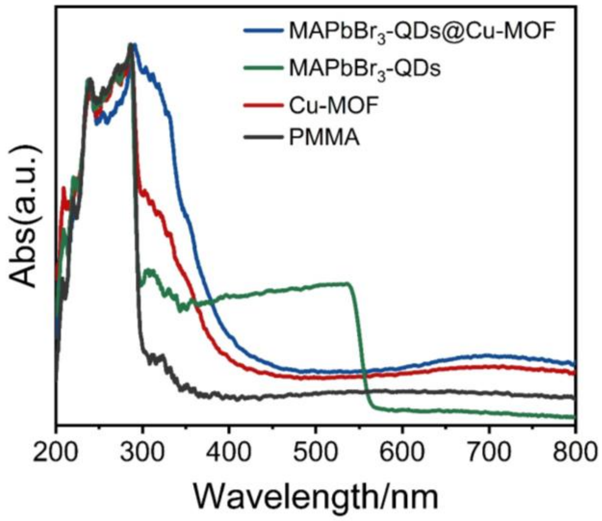

Figure S16. Solid-state UV-Vis absorption spectra of PMMA, Cu-MOF, $MAPbBr_3$-QDs, and $MAPbBr_3$-QDs@Cu-MOF films.

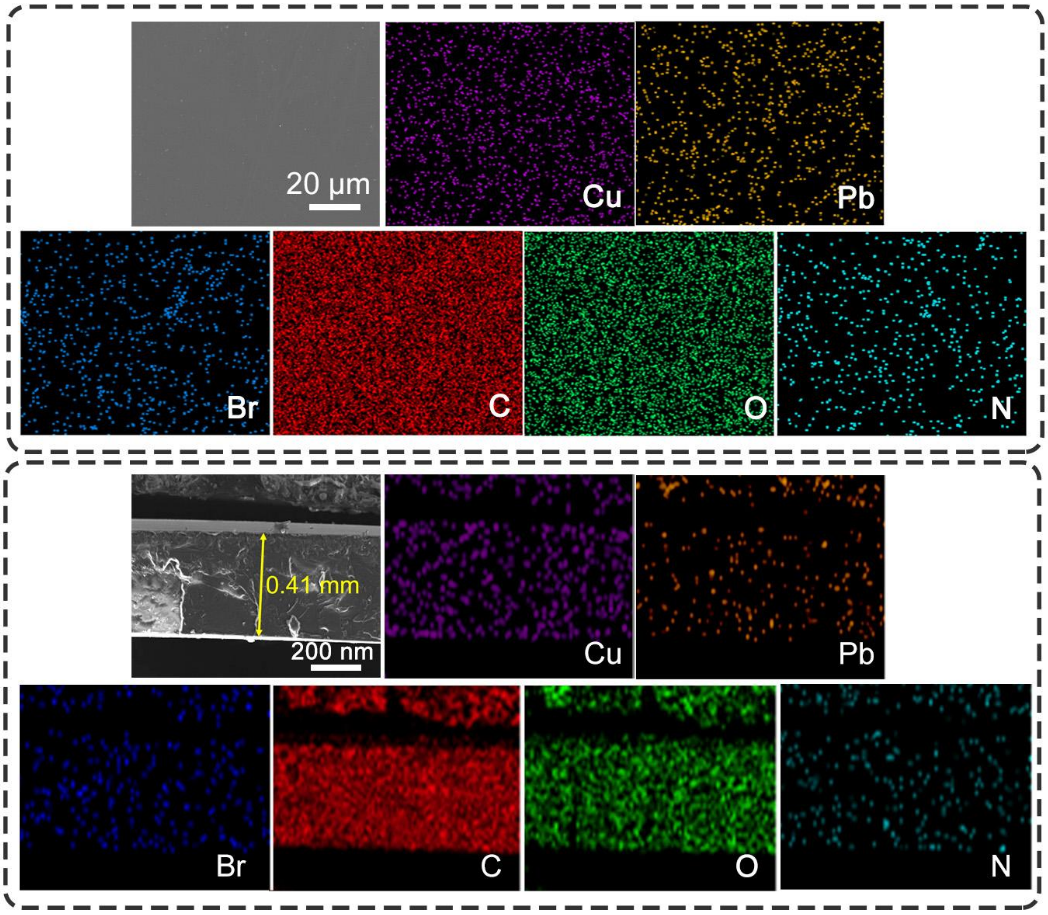

Figure S17. Surface and cross-sectional SEM images of $MAPbBr_3$-QDs@Cu-MOF/PMMA, as well as element distribution maps of Cu, Pb, Br, C, O, N.

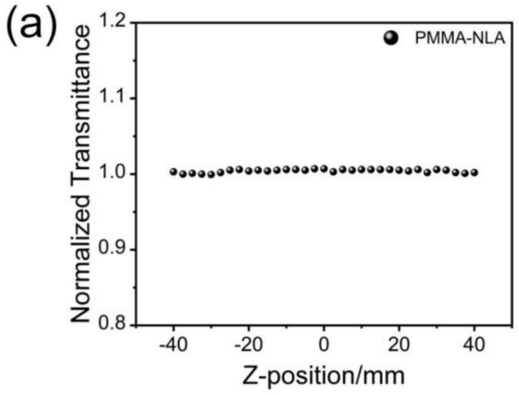

Figure S18. The NLA data and NLR data of pure PMMA film at a pulsed wavelength of 532 nm.

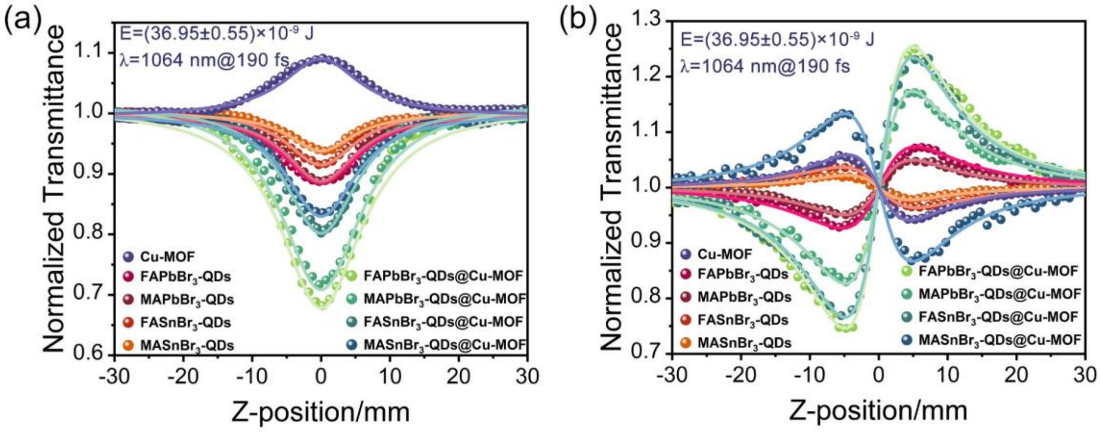

Figure S19. (a) NLA and (b) NLR results of Cu-MOF, $MAPbBr_3$-QDs, $FAPbBr_3$-QDs, $MASnBr_3$-QDs, $FASnBr_3$-QDs, $FAPbBr_3$-QDs@Cu-MOF, $MAPbBr_3$-QDs@Cu-MOF, $MASnBr_3$-QDs@Cu-MOF and $FASnBr_3$-QDs@Cu-MOF at a pulsed wavelength of 1064 nm.

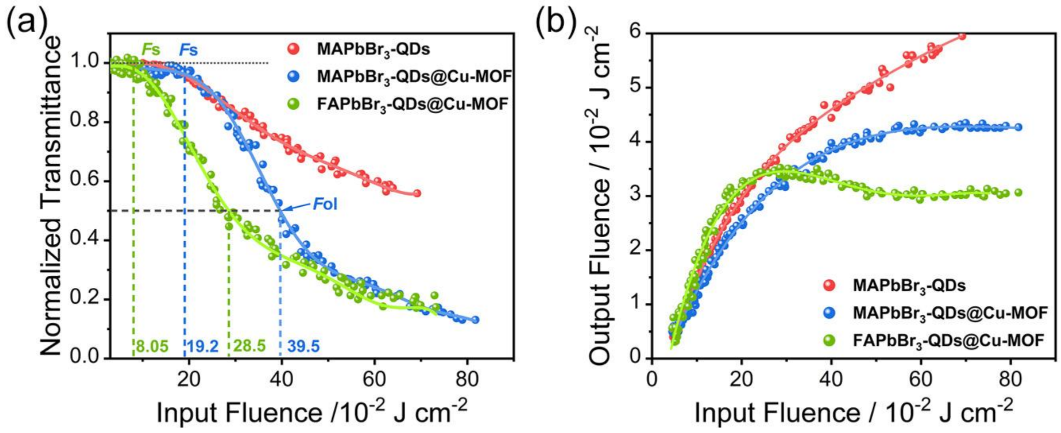

Figure S20. (a) Normalized transmittance of the $MAPbBr_3$-QDs, $MAPbBr_3$-QDs@Cu-MOF, and $FAPbBr_3$-QDs@Cu-MOF at the beam waist is related to the input fluence; (b) Output fluence of the $MAPbBr_3$-QDs, $MAPbBr_3$-QDs@Cu-MOF, and $FAPbBr_3$-QDs@Cu-MOF at the beam waist is related to the input fluence.

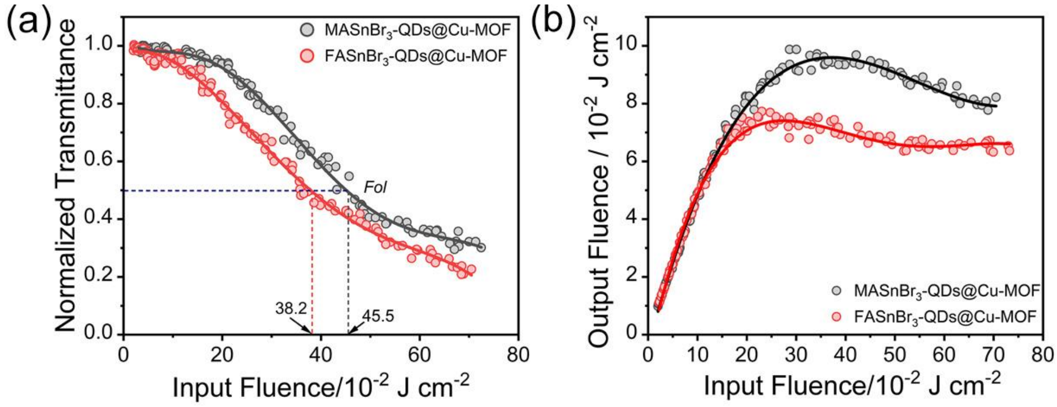

Figure S21. (a) Normalized transmittance of the $MASnBr_3$-QDs@Cu-MOF and $FASnBr_3$-QDs@Cu-MOF at the beam waist is related to the input fluence; (b) Output fluence of the $MASnBr_3$-QDs@Cu-MOF and $FASnBr_3$-QDs@Cu-MOF at the beam waist is related to the input fluence.

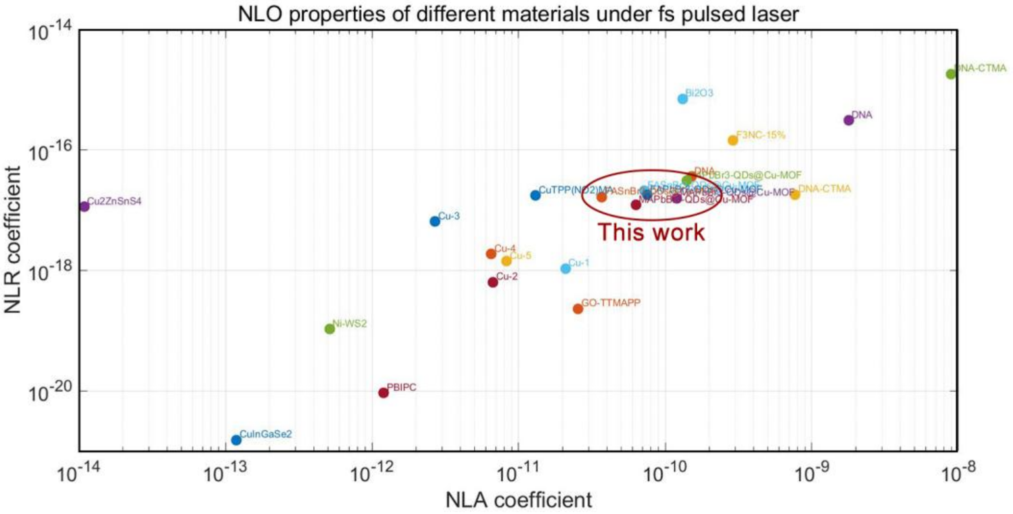

Figure S22. NLO properties of different materials under femtosecond pulsed laser.

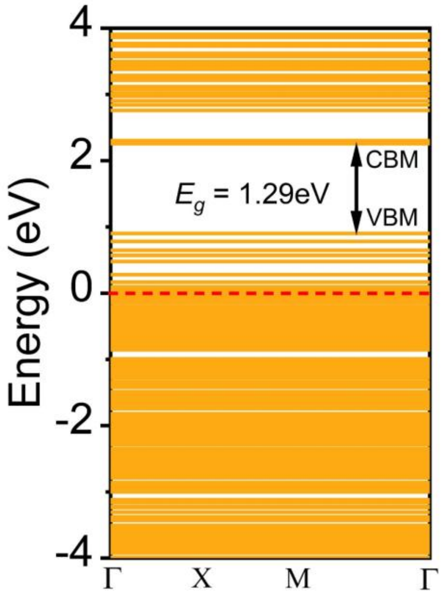

Figure S23. Band structure of Cu-MOF.

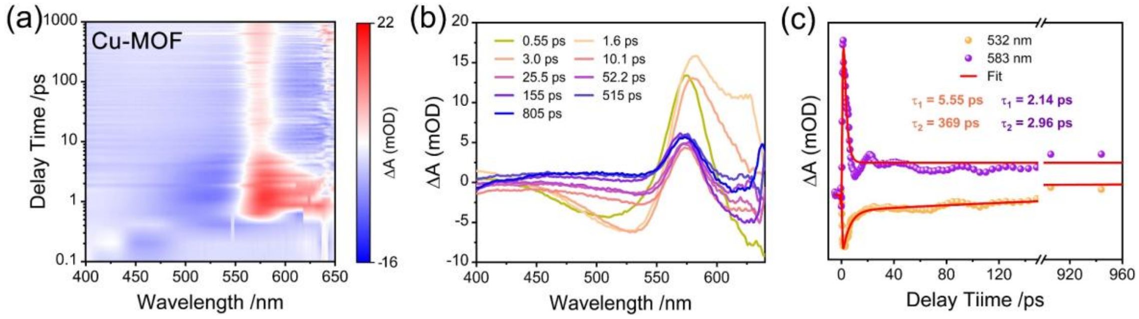

Figure S24. (a) Two-dimensional pseudocolor TAS maps of Cu-MOF; (b) TAS of Cu-MOF on the time (0.55 ps - 805 ps) scales upon excitation at 350 nm; (c) Time-resolved TAS of Cu-MOF at different wavelengths.

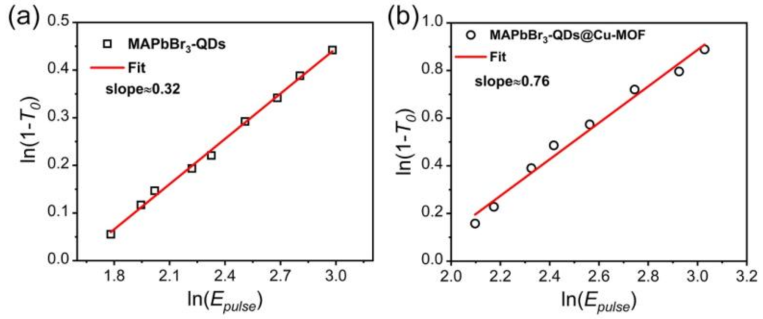

Figure S25. Plot of ln(1-T_0) versus ln(E_{pulse}) to determine the order of optical nonlinearity of (a) $MAPbBr_3$-QDs and (b) $MAPbBr_3$-QDs@Cu-MOF.

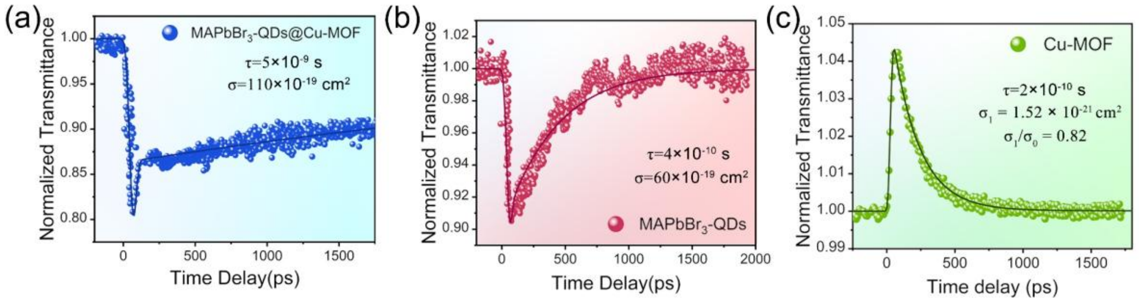

Figure S26. The transient absorption spectrum of (a) $MAPbBr_3$-QDs@Cu-MOF, (b) $MAPbBr_3$-QDs, and (c) Cu-MOF.

To elucidate the photogenerated carrier dynamics in $MAPbBr_3$-QDs@Cu-MOF, Carrier dynamics

in $MAPbBr_3$-QDs and $MAPbBr_3$-QDs@Cu-MOF were further investigated using time-resolved pump-probe spectroscopy. The materials were excited using pump pulses centered at 532 nm, and the dynamics of the photogenerated carriers were investigated using probe pulses with a time resolution of 21 ps (after numerical deconvolution) at various time delays.[9-11] When the samples were excited by pump pulses with energies above the bandgap, higher-energy electrons and holes were generated. Following optical excitation, ultrafast scattering processes (such as carrier-carrier or carrier-optical phonon scattering, occurring on the timescale of a few picoseconds) lead to carrier thermalization. In $MAPbBr_3$-QDs@Cu-MOF, intraband relaxation (vibrational relaxation) occurs within a timespan of a few to tens of picoseconds. Additionally, the presence of surface defects increases the capture of charge carriers. The recovery of the ground-state bleach (GSB) signal originates from the filling of the valence and conduction band states. The fast component of the excited-state absorption (ESA) is attributed to the relaxation of hot electrons in the conduction band, while the fast component of GSB is attributed to the relaxation of hot electrons and holes in the valence band. The slow components are associated with electron capture in ESA and electron and hole capture in GSB.[12, 13] The transient absorption spectrum of $MAPbBr_3$-QDs@Cu-MOF is shown in Figure S26a. Before zero delay, the pump light has not yet reached $MAPbBr_3$-QDs@Cu-MOF, and the probe light energy is insufficient to excite electrons in the medium for inter-level transitions. At zero delay, electrons in the VB absorb photon energy and transition to high-energy states in the CB. Within the delay range of 70-130 ps, the normalized transmittance increases rapidly, indicating that some electrons in $MAPbBr_3$-QDs and Cu-MOF directly relax back to the VB. After approximately 130 ps of delay, the signal exhibits a flattening behavior, which is primarily due to the higher position of the CB in $MAPbBr_3$-QDs compared to Cu-MOF. As a result, some high-energy electrons quickly transfer from $MAPbBr_3$-QDs to Cu-MOF through interfacial interactions, thereby extending the carrier transport path and range. In contrast, in $MAPbBr_3$, electrons in higher energy states relax back to the VB without undergoing other transport pathways. As the time delay increases, without additional energy input, the generated free carriers will eventually recombine over time, leading to the recovery of linear absorption. Fitting the experimental data gives the carrier lifetime (τ) of $MAPbBr_3$-QDs@Cu-MOF as 5×10^{-9} s and the carrier absorption cross-section (σ) as 110×10^{-19} cm^2. Compared to pure $MAPbBr_3$-QDs ($\tau = 4 \times 10^{-9}$ s, $\sigma = 60 \times 10^{-19}$ cm^2) (Figure S26b),[14] the carrier absorption cross-section is enhanced by a factor of 1.83. This indicates that the increase in carrier absorption cross-section strengthens the ability of

carriers to absorb incident light, thereby triggering carrier accumulation effects, affecting carrier lifetime and electron relaxation dynamics, and ultimately enhancing the third-order NLO response of $MAPbBr_3$. In the TA spectrum of Cu-MOF (Figure S26c), a positive absorption peak lasting approximately 81 ps was observed, indicating the occurrence of a photobleaching process. This process can be attributed to the Pauli blocking effect caused by the transition of VB electrons to the CB. Subsequently, the normalized transmittance continuously decreases and eventually recovers to 1 over a period of approximately 860 ps. This process involves the relaxation of hot electrons and holes, ultimately leading to the recombination of electrons and holes, and restoring the system to a state of equilibrium.

Table S1. Third-order NLO parameters of Cu-MOF, $MAPbBr_3$-QDs, $MAPbBr_3$-QDs@Cu-MOF-5.7, $MAPbBr_3$-QDs@Cu-MOF-9.1, $MAPbBr_3$-QDs@Cu-MOF-17.6, $MAPbBr_3$-QDs@Cu-MOF-35.4, and $MAPbBr_3$-QDs@Cu-MOF-38.9 under 532 nm laser irradiation.

	E (10^{-6} J)	T	L	N.T.	β_{eff} (10^{-12} m/W)	n_2 (10^{-19} m^2/W)
Cu-MOF	1.29	0.61	0.38	1.33	-200	-60
$MAPbBr_3$-QDs	1.25	0.64	0.38	0.81	82	70
$MAPbBr_3$-QDs@Cu-MOF-5.7	1.27	0.76	0.41	0.74	125	65
$MAPbBr_3$-QDs@Cu-MOF-9.1	1.27	0.70	0.41	0.63	185	125
$MAPbBr_3$-QDs@Cu-MOF-17.6	1.24	0.52	0.38	0.55	265	220
$MAPbBr_3$-QDs@Cu-MOF-35.4	1.26	0.58	0.40	0.43	280	245
$MAPbBr_3$-QDs@Cu-MOF-38.9	1.29	0.61	0.40	0.61	195	130

Table S2. Third-order NLO parameters of Cu-MOF, $MAPbBr_3$-QDs, $FAPbBr_3$-QDs, $MASnBr_3$-QDs, $FASnBr_3$-QDs, $MAPbBr_3$-QDs@Cu-MOF, $FAPbBr_3$-QDs@Cu-MOF, $MASnBr_3$-QDs@Cu-MOF, and $FASnBr_3$-QDs@Cu-MOF under 532 nm laser irradiation.

	E (10^{-6} J)	T	L	N.T.	β_{eff} (10^{-12} m/W)	n_2 (10^{-19} m^2/W)
Cu-MOF	1.29	0.61	0.38	1.33	-200	-60
$MAPbBr_3$-QDs	1.25	0.64	0.38	0.81	82	70
$FAPbBr_3$-QDs	1.34	0.71	0.42	0.88	46	37
$MASnBr_3$-QDs	1.27	0.80	0.40	0.90	32	-90
$FASnBr_3$-QDs	1.22	0.72	0.39	0.86	57	-180
$MAPbBr_3$-QDs@Cu-MOF	1.26	0.58	0.40	0.43	280	245
$FAPbBr_3$-QDs@Cu-MOF	1.32	0.52	0.42	0.35	360	160
$MASnBr_3$-QDs@Cu-MOF	1.25	0.55	0.41	0.62	160	-150
$FASnBr_3$-QDs@Cu-MOF	1.28	0.52	0.40	0.50	170	230

Table S3. Third-order NLO parameters of Cu-MOF, $MAPbBr_3$-QDs, $MAPbBr_3$-QDs@Cu-MOF-5.7, $MAPbBr_3$-QDs@Cu-MOF-9.1, $MAPbBr_3$-QDs@Cu-MOF-17.6, $MAPbBr_3$-QDs@Cu-MOF-35.4, and $MAPbBr_3$-QDs@Cu-MOF-38.9 under 900 nm laser irradiation.

	E (10^{-9} J)	T	L	N.T.	β_{eff} (10^{-12} m/W)	n_2 (10^{-19} m^2/W)
Cu-MOF	40.2	0.65	0.38	1.18	-42	-90
$MAPbBr_3$-QDs	40.5	0.69	0.38	0.84	20	80
$MAPbBr_3$-QDs@Cu-MOF-5.7	40.0	0.80	0.41	0.86	13	50
$MAPbBr_3$-QDs@Cu-MOF-9.1	39.8	0.75	0.41	0.80	22	65
$MAPbBr_3$-QDs@Cu-MOF-17.6	40.5	0.65	0.40	0.70	47	88
$MAPbBr_3$-QDs@Cu-MOF-35.4	39.5	0.63	0.38	0.49	120	158
$MAPbBr_3$-QDs@Cu-MOF-38.9	40.2	0.67	0.40	0.67	50	105

Table S4. Third-order NLO parameters of Cu-MOF, $MAPbBr_3$-QDs, $FAPbBr_3$-QDs, $MASnBr_3$-QDs, $FASnBr_3$-QDs, $MAPbBr_3$-QDs@Cu-MOF, $FAPbBr_3$-QDs@Cu-MOF, $MASnBr_3$-QDs@Cu-MOF, and $FASnBr_3$-QDs@Cu-MOF under 900 nm laser irradiation.

	E (10^{-9} J)	T	L	N.T.	β_{eff} (10^{-12} m/W)	n_2 (10^{-19} m^2/W)
Cu-MOF	40.2	0.65	0.38	1.18	-42	-90
$MAPbBr_3$-QDs	40.5	0.69	0.38	0.84	20	80
$FAPbBr_3$-QDs	39.8	0.76	0.42	0.82	22	123
$MASnBr_3$-QDs	41.2	0.83	0.40	0.88	13	-59
$FASnBr_3$-QDs	40.0	0.78	0.39	0.85	18	-105
$MAPbBr_3$-QDs@Cu-MOF	39.5	0.63	0.40	0.49	120	158
$FAPbBr_3$-QDs@Cu-MOF	40.0	0.60	0.42	0.41	140	310
$MASnBr_3$-QDs@Cu-MOF	40.3	0.62	0.41	0.62	57	-142
$FASnBr_3$-QDs@Cu-MOF	39.4	0.61	0.40	0.58	72	210

Table S5. Third-order NLO parameters of Cu-MOF, $MAPbBr_3$-QDs, $FAPbBr_3$-QDs, $MASnBr_3$-QDs, $FASnBr_3$-QDs, $MAPbBr_3$-QDs@Cu-MOF, $FAPbBr_3$-QDs@Cu-MOF, $MASnBr_3$-QDs@Cu-MOF, and $FASnBr_3$-QDs@Cu-MOF under 1064 nm laser irradiation.

	E (10^{-9} J)	T	L	N.T.	β_{eff} (10^{-12} m/W)	n_2 (10^{-19} m^2/W)
Cu-MOF	36.5	0.67	0.38	1.09	-28	-40
$MAPbBr_3$-QDs	37.2	0.72	0.38	0.89	19	35
$FAPbBr_3$-QDs	36.9	0.79	0.42	0.88	21	50
$MASnBr_3$-QDs	37.2	0.85	0.40	0.94	8	-12
$FASnBr_3$-QDs	37.0	0.80	0.39	0.91	14	-27
$MAPbBr_3$-QDs@Cu-MOF	37.0	0.67	0.40	0.71	63	122
$FAPbBr_3$-QDs@Cu-MOF	37.0	0.62	0.42	0.68	75	180
$MASnBr_3$-QDs@Cu-MOF	36.9	0.65	0.41	0.83	28	-95
$FASnBr_3$-QDs@Cu-MOF	37.4	0.64	0.40	0.80	37	165

Table S6. The NLA and NLR coefficients of different materials under femtosecond pulsed laser and the corresponding references.

Materials	Laser parameter	β (m/W)	n_2 (m^2/W)	references
$CuInGaSe_2$	800 nm@90 fs@1kHz	$(1.191 \pm 0.485) \times 10^{-13}$	$(1.526 \pm 0.249) \times 10^{-21}$	S15
GO-TTMAPP	800 nm@100 fs@80 MHz	2.55×10^{-11}	2.32×10^{-19}	S16
F_3NC-15%	800 nm@150 fs@80 MHz	$(2.90 \pm 0.09) \times 10^{-10}$	$(1.437 \pm 0.015) \times 10^{-16}$	S17
Cu_2ZnSnS_4	800 nm@90 fs@1 kHz	1.090×10^{-14}	1.146×10^{-17}	S18
Ni-WS_2	800 nm@35 fs@1 kHz	$(5.13 \pm 0.26) \times 10^{-13}$	$(1.06 \pm 0.053) \times 10^{-19}$	S19
Ta_2C	800 nm@100 fs @1 kHz	-4.2×10^{-12}	-	S20
	475 nm@100 fs@1 kHz	-1.5×10^{-12}	-	
	1550 nm@100 fs@1 kHz	-1.3×10^{-12}	-	
Bi_2O_3	800 nm@120 fs@1 kHz	1.31×10^{-10}	7.1×10^{-16}	S21
PBIPC	800 nm@150 fs@80 kHz	1.20×10^{-12}	9.2×10^{-21}	S22
CuTPP(NO_2)MA	800 nm@120 fs@1 kHz	1.303×10^{-11}	1.75×10^{-17}	S23
$CuFeTe_2$	800 nm@100 fs@2 kHz	-1.5×10^{-12}	-1.66×10^{-16}	S24
graphene	1150 nm@100 fs@1 kHz	3.8×10^{-12}	-5.5×10^{-14}	S25
o-CB (1-1)	650 nm@200 fs@10 kHz	2.0×10^{-13}	-	S26
o-CB (1-2)		2.3×10^{-13}	-	
o-CB (2-1)		7.0×10^{-13}	-	
DNA film	800 nm@110 fs@79.8 MHz	1.51×10^{-10}	3.63×10^{-17}	S27
DNA-CTMA film		7.71×10^{-10}	1.81×10^{-17}	
DNA film	1570 nm@110 fs@79.8 MHz	1.78×10^{-9}	3.11×10^{-16}	
DNA-CTMA film		8.89×10^{-9}	1.82×10^{-15}	
C1	800 nm@190 fs@20 Hz	6×10^{-14}	-	S28
C2		9×10^{-14}	-	
SiNS-dodecene	800 nm @50 fs@10 Hz	-4.00×10^{-13}	1.33×10^{-19}	S29
Cu-1	800 nm @120 fs@1 kHz	2.09×10^{-11}	1.08×10^{-18}	S30

Cu-2		6.7×10^{-12}	6.4×10^{-19}	
Cu-3		2.7×10^{-12}	6.45×10^{-18}	
Cu-4		6.5×10^{-12}	1.88×10^{-18}	
Cu-5		8.3×10^{-12}	1.43×10^{-18}	
$MAPbBr_3$-QDs@Cu-MOF	900 nm@190 fs@20Hz	1.20×10^{-10}	1.58×10^{-17}	This work
$FAPbBr_3$-QDs@Cu-MOF		1.40×10^{-10}	3.10×10^{-17}	
$MASnBr_3$-QDs@Cu-MOF		5.7×10^{-11}	-1.42×10^{-17}	
$FASnBr_3$-QDs@Cu-MOF		7.2×10^{-11}	2.10×10^{-17}	
$MAPbBr_3$-QDs@Cu-MOF	1064 nm@190 fs@20Hz	6.3×10^{-11}	1.22×10^{-17}	
$FAPbBr_3$-QDs@Cu-MOF		7.5×10^{-11}	1.80×10^{-17}	
$MASnBr_3$-QDs@Cu-MOF		2.8×10^{-11}	-9.5×10^{-18}	
$FASnBr_3$-QDs@Cu-MOF		3.7×10^{-11}	1.65×10^{-17}	

References

S1. Sun, Y.; Xu, W.; Lang, F.; Wang, H.; Pan, F.; Hou, H., Transformation of SBUs and Synergy of MOF Host-Guest in Single Crystalline State: Ingenious Strategies for Modulating Third-Order NLO Signals. *Small* **2024,** *20* (4), 2305879.

S2. Kresse, G., Hafner, J. Ab initio molecular dynamics for open-shell transition metals. *Phys. Rev. B Condens. Matter* **1993**, *48*, 13115-13118.

S3. Kresse, G. F. l., J. Efficient iterative schemes for ab initio total-energy calculations using a plane-wave basis set. *Phys. Rev. B Condens. Matter* **1996**, *54*, 169-185.

S4. John P. Perdew, K. B., and Matthias Ernzerhof. Generalized gradient approximation made simple. *Phys. Rev. Lett* **1996**, *77*.

S5. Kohn, W. & Sham, L. J. Self-Consistent Equations Including Exchange and Correlation Effects. *Phys. Rev.* **1965**, *140*, A1133-A1138.

S6. Grimme, S., Antony, J., Ehrlich, S., Krieg, H. A consistent and accurate ab initio parametrization of density functional dispersion correction (DFT-D) for the 94 elements H-Pu. *J. Chem. Phys.* **2010**, *132*, 154104.

S7. Wang, V., Xu, N., Liu, J.-C., Tang, G. & Geng, W.-T. VASPKIT: A user-friendly interface facilitating high-throughput computing and analysis using VASP code. *Comput Phys Commun* **2021**, *267*, 108033.

S8. Momma, K. & Izumi, F. VESTA: a three-dimensional visualization system for electronic and structural analysis. *J. Appl. Crystallogr.* **2008**, *41*, 653-658.

S9. Wang, R.; Xue, J.; Wang, K. L.; Wang, Z. K.; Luo, Y.; Fenning, D.; Xu, G.; Nuryyeva, S.; Huang, T.; Zhao, Y.; Yang, J. L.; Zhu, J.; Wang, M.; Tan, S.; Yavuz, I.; Houk, K. N.; Yang, Y., Constructive molecular configurations for surface-defect passivation of perovskite photovoltaics. *Science* **2019,** *366* (6472), 1509-1513.

S10. Ruan, R.; Wu, X.; Li, X.; Jia, J.; Fang, Y.; Chen, Y.; Wu, Q.; Song, Y., Giant excited-state absorption in resonant absorption band and intensity-depend ultrafast sign switching of nonlinear absorption in azonaphthalene compound. *Opt. Mater.* **2024,** *154*, 115667.

S11. Zou, Y.; Yu, Z.; Ma, H.; Zhao, C.; Wang, B.; Li, R.; Li, X.; Yang, J.; Li, F.; Yu, W., Deciphering the Carrier Transport Properties in Two-Dimensional Perovskites via Surface-Enhanced Raman Scattering. *Small* **2021,** *17* (49), 2103756.

S12. Tailor, N. K.; Mishra, S.; Sharma, T.; De, A. K.; Satapathi, S., Cation-Dependent Hot Carrier Cooling in the Lead-Free Bismuth Halide $A_3Bi_2I_9$ (A = FA, MA, and Cs) Perovskite. *J. Phys. Chem. C* **2021,** *125* (18), 9891-9898.

S13. Kawai, H.; Giorgi, G.; Marini, A.; Yamashita, K., The Mechanism of Slow Hot-Hole Cooling in Lead-Iodide Perovskite: First-Principles Calculation on Carrier Lifetime from Electron-Phonon Interaction. *Nano Lett.* **2015,** *15* (5), 3103-3108.

S14. Kalanoor, B. S.; Gouda, L.; Gottesman, R.; Tirosh, S.; Haltzi, E.; Zaban, A.; Tischler, Y. R., Third-Order Optical Nonlinearities in Organometallic Methylammonium Lead Iodide Perovskite Thin Films. *ACS Photonics* **2016,** *3* (3), 361-370.

S15. Kılıç, H. Ş.; Yiğit Gezgin, S.; Üzüm, Ö.; Gündoğdu, Y., Third order nonlinear optical properties of copper indium gallium selenide (CIGS) nanocrystal thin films. *Appl. Phys. B* **2022,** *128* (6), 105.

S16. Mousa, K.; Samad, F. A.; Mohamed, T.; El-Khouly, M. E., Cationic porphyrin-functionalized

graphene oxide: A novel platform for ultrafast femtosecond nonlinear optical limiting. *J. Colloid Interface Sci.* **2025,** *692*, 137549.
S17. Ekbote, A. N.; Maidur, S. R.; Jahagirdar, J. R.; Patil, P. S.; Soma, V. R., Femtosecond nonlinear optical investigations of nitro chalcones-doped PMMA thin films for optical limiting and photonic applications. *Mater. Today Commun.* **2023,** *37*, 107240.
S18. Kılıç, H. Ş.; Gündoğdu, Y.; Kılıç, S.; Yiğit Gezgin, S., Nonlinear optical properties of Cu_2ZnSnS_4 nanocrystal thin films and its constituents thin films. *Opt. Quantum Electron.* **2021,** *53* (1), 19.
S19. Konda, S. R.; Rajan, R. A.; Singh, S.; Ganeev, R. A.; Soma, V. R.; Srivastava, A.; Venkatesh, M.; Guo, C.; Li, W., Influence of embedded NiO-nanoparticles on the nonlinear absorption of tungsten disulfide nanolayers. *Opt. Mater.* **2023,** *138*, 113657.
S20. Guo, J.; Liu, Z.; Wageh, S.; Al-Hartomy, O. A.; Al-Sehemi, A. G.; Ge, Y.; He, W.; Wei, S.; Bao, W.; Zhang, H., Ta_2C MXene: Nonlinear optical properties and application in femtosecond fiber laser. *Opt. Laser Technol.* **2023,** *161*, 109178.
S21. Acharyya, J. N.; Desai, N. R.; Gangineni, R. B.; Vijaya Prakash, G., Effect of Photonic Cavity Interactions on Femtosecond Multiphoton Optical Nonlinear Absorptions from Bi2O3-Based One-Dimensional Photonic Crystal. *ACS Photonics* **2022,** *9* (6), 2092-2100.
S22. Bhattacharya, S.; Biswas, C.; Raavi, S. S. K.; Venkata Suman Krishna, J.; Koteshwar, D.; Giribabu, L.; Venugopal Rao, S., Optoelectronic, femtosecond nonlinear optical properties and excited state dynamics of a triphenyl imidazole induced phthalocyanine derivative. *RSC Adv.* **2019,** *9* (63), 36726-36741.
S23. Bulbul, A. S.; Chaudhri, N.; Shanu, M.; Acharyya, J. N.; Vijaya Prakash, G.; Sankar, M., Unsymmetrically β-Functionalized π-Extended Porphyrins: Synthesis, Spectral, Electrochemical Redox Properties, and Their Utilization as Efficient Two-Photon Absorbers. *Inorg. Chem.* **2022,** *61* (26), 9968-9982.
S24. Chen, Z.; Fan, W.; Xu, D.; Dong, Y.; Chen, Z.; Gu, Z.; Fang, M.; Xiao, S.; Zhu, M.; He, J., Origin of the Efficient Nonlinear Optical Response of Two-Dimensional Layered $CuFeTe_2$ Nanosheets. *J. Phys. Chem. Lett.* **2022,** *13* (33), 7770-7778.
S25. Lu, S.; Ge, Y.; Sun, Z.; Huang, Z.; Cao, R.; Zhao, C.; Wen, S.; Fan, D.; Li, J.; Zhang, H., Ultrafast nonlinear absorption and nonlinear refraction in few-layer oxidized black phosphorus. *Photon. Res.* **2016,** *4* (6), 286-292.
S26. Feng, W.; Liu, K.; Zang, J.; Wang, G.; Miao, R.; Ding, L.; Liu, T.; Kong, J.; Fang, Y., Flexible and Transparent Oligothiophene-o-Carborane-Containing Hybrid Films for Nonlinear Optical Limiting Based on Efficient Two-Photon Absorption. *ACS Appl. Mater. Interfaces* **2021,** *13* (24), 28985-28995.
S27. Khazaeinezhad, R.; Hosseinzadeh Kassani, S.; Paulson, B.; Jeong, H.; Gwak, J.; Rotermund, F.; Yeom, D.-I.; Oh, K., Ultrafast nonlinear optical properties of thin-solid DNA film and their application as a saturable absorber in femtosecond mode-locked fiber laser. *Sci Rep* **2017,** *7* (1), 41480.
S28. Shen, L.; Li, Z.; Wu, X.; Zhou, W.; Yang, J.; Song, Y., Ultrafast broadband nonlinear optical properties and excited-state dynamics of two bis-chalcone derivatives. *RSC Adv.* **2020,** *10* (26), 15199-15205.
S29. Stathis, A.; Stavrou, M.; Papadakis, I.; Mock, J.; Kloberg, M. J.; Becherer, M.; Lyuleeva-Husemann, A.; Couris, S., Silicon Nanosheets: A Promising 2D Material with Strong Ultrafast Nonlinear Optical Response. *J. Phys. Chem. C* **2021,** *125* (33), 18510-18516.
S30. Yadav, I.; Shanu, M.; Acharyya, J. N.; Prakash, G. V.; Sankar, M., Ultrafast Dynamics and Strong

Two-Photon Absorption Properties of Nonplanar β-Functionalized “Push–Pull” Copper Corroles with a Mixed Substituent Pattern. *Inorg. Chem.* **2022,** *61* (48), 19289-19301.
